# Utilizing Bayesian inference in accelerated testing models under constant stress via ordered ranked set sampling and hybrid censoring with practical validation

**DOI:** 10.1038/s41598-024-64718-w

**Published:** 2024-06-22

**Authors:** Atef F. Hashem, Naif Alotaibi, Salem A. Alyami, Mohamed A. Abdelkawy, Mohamed A. Abd Elgawad, Haitham M. Yousof, Alaa H. Abdel-Hamid

**Affiliations:** 1 Department of Mathematics and Statistics, College of Science, Imam Mohammad Ibn Saud Islamic University (IMSIU), Riyadh 11432, Saudi Arabia; 2 Department of Statistics, Mathematics and Insurance, Benha University, Benha, 13518 Egypt; 3 Mathematics and Computer Science Department, Faculty of Science, Beni-Suef University, Beni-Suef, 62511 Egypt; 4 Department of Mathematics, Faculty of Science, Benha University, Benha, 13518 Egypt

**Keywords:** Accelerated life-testing, Half-logistic model, Type-I hybrid censoring, Ordered ranked set sampling, Bayesian estimation, Simulation, Applied mathematics, Statistics

## Abstract

This research investigates the application of the ordered ranked set sampling (ORSSA) procedure in constant-stress partially accelerated life-testing (CSPALTE). The study adopts the assumption that the lifespan of a specific item under operational stress follows a half-logistic probability distribution. Through Bayesian estimation methods, it concentrates on estimating the parameters, utilizing both asymmetric loss function and symmetric loss function. Estimations are conducted using ORSSAs and simple random samples, incorporating hybrid censoring of type-I. Real-world data sets are utilized to offer practical context and validate the theoretical discoveries, providing concrete insights into the research findings. Furthermore, a rigorous simulation study, supported by precise numerical calculations, is meticulously conducted to gauge the Bayesian estimation performance across the two distinct sampling methodologies. This research ultimately sheds light on the efficacy of Bayesian estimation techniques under varying sampling strategies, contributing to the broader understanding of reliability analysis in CSPALTE scenarios.

## Introduction

life-testing serves the purpose of scrutinizing the failure times of test units acquired under typical operational conditions. In contemporary product design, where longevity is a key focus, gathering failure data under regular circumstances can prove challenging or even unfeasible. In such scenarios, subjecting items to stress levels higher than their manufacturing specifications becomes necessary to acquire data about their failure times. This type of life test conducted under challenging conditions is known as accelerated life-testing (ALTE). The failure times observed during ALTE can be utilized to ascertain the life characteristics of the units under normal usage conditions.

Various models, such as the inverse power and the Arrhenius models are employed to introduce acceleration in experiments. The choice of the acceleration model depends on the type of stress (voltage, pressure, or temperature) the researcher aims to apply. Typically, the Arrhenius model is used for thermal stresses, while the inverse power model is preferred for non-thermal stresses. ALTE comes in different forms, including constant-stress ALTE (CSALTE), see^1–3^, step-stress ALTE (SSALTE)^4–6^, and progressive-stress ALTE (PSALTE), see^7–10^. In CSALTE, stress remains constant throughout the entire experiment, while it gradually increases at specific intervals in SSALTE. Conversely, stress in PSALTE is a function of time, increasing progressively. ALTEs can also be categorized, based on the number of stress levels, into two types: Simple ALTE, which has only two stress levels, and multiple ALTE, which incorporates more than two levels of stress. For more details on types of ALTEs, see^11–13^.

Several authors have studied Bayes and maximum likelihood estimation techniques under ALTEs. AL-Hussaini and Abdel-Hamid^1,2^ considered finite mixtures of distributions in the context of CSPALTE. Abdel-Hamid^14^ focused on CSPALTE in cases where the lifespan of units operating under specific conditions follows the Burr XII distribution. In another study, Mohie El-Din et al.^3^ devised a Bayesian estimation approach for CSPALTE, extending it to encompass the exponential distribution within a framework of progressive censoring. Furthermore, the regression analysis is considered another technique in estimating the parameters under consideration, see, for example, Wang et al.^15^. Xu et al.^16^ employed Bayesian methodology to assess the reliability of permanent magnet brakes when dealing with small sample sizes.

In the realm of estimation, Hassan et al.^17^ addressed the challenge of estimating parameters for the CSPALTE competing failure model, assuming a Weibull distribution and considering both types I and II censoring. Similarly, Abdullah et al.^18^ delved into the task of estimating parameters of Fréchet distribution within the CSPALTE framework, concentrating on type-I censoring.

### Half-logistic model

The half-logistic model is a probability distribution that is related to the logistic probability distribution. It is commonly used in various fields, including statistics and economics, to model situations where data exhibit certain characteristics. It can also be useful in survival analysis and reliability engineering when dealing with skewed data and non-negative lifetimes. Moreover, it has been applied as a life-testing model by several authors, see, for example^19,20^.

Respectively, the cumulative distribution function (CDFU) and its corresponding probability density function (PDFU) of the half-logistic random variable (RV) *T* (with realization *t*) are provided as follows:

1 $$\begin{aligned}{} & {} H_1(t)=H_1(t|T,\eta )=\displaystyle \frac{1- e^{-t/\eta }}{1+ e^{-t/\eta }}| t>0, \; (\eta >0), \end{aligned}$$

2 $$\begin{aligned}{} & {} h_1(t)=h_1(t|T,\eta )=\displaystyle \frac{2 e^{-t/\eta }}{\eta \left( 1+ e^{-t/\eta }\right) ^{2}}| t>0,\; (\eta >0). \end{aligned}$$

### Type-I hybrid censoring

In the realm of reliability studies and survival analysis, type-I hybrid censoring emerges as a strategic approach that amalgamates the principles of both type-I and type-II censoring. Let’s dissect these concepts to gain a comprehensive understanding.

Type-I censorship involves terminating an experiment at a pre-defined time, without considering whether the event of interest (such as failure) has occurred or not. Conversely, type-II censoring concludes the investigation after a specific number of incidents (failures) have been observed. Individuals or groups that are still ongoing at the conclusion of the study, having not experienced the event, are deemed censored.

Now, in the landscape of type-I hybrid censoring, both these censoring processes are intricately intertwined. In a type-I hybrid scenario, the study concludes either when a predetermined time limit is reached or when a specified number of events have transpired. This dual criterion for concluding the study is denoted as $T^{\star } = \min (T_{s:m}, \tau )$, where $T_{s:m}$ represents the time when the specified count *s* of failures occurs, and $\tau$ signifies the predetermined time limit.

In practical terms, when subjecting a batch of *m* units to testing, the individual lifetimes of these units are considered as independent and identically distributed random variables, denoted by $T_{1}, T_{2},\ldots , T_{m}$. The testing process dynamically concludes based on the occurrence of either a specified count of failures or the elapse of a predefined time. This nuanced methodology allows investigators to optimize study time, striking a balance between the benefits of type-I and type-II censorship, offering flexibility and efficiency in reliability analysis.

Hybrid censoring has gained increased prominence, prompting numerous researchers to explore statistical analysis techniques for different distributions within the realm of hybrid censoring in reliability studies. Noteworthy contributors to this area include^21–25^, among other scholars.

### Ranked set sampling

The primary method for gathering data through sampling is known as SRSA, which involves randomly selecting sampling units. Assigning ranks to various sampling units without the need for actual measurements can be relatively straightforward and cost-effective in various fields (e.g., fisheries and medical research). This is especially useful when it would be expensive and time-consuming to measure the variable of interest directly. To obtain more representative samples from the wider population in such circumstances, sampling designs that emphasise ranks can be used, which will ultimately increase the efficacy of statistical analysis. Ranked set sampling (RSSA) was originally suggested by McIntyre^26^, and multiple studies, whether through numerical or theoretical means, have illustrated the advantages of employing RSSA-based statistical methods compared to their counterparts in the SRSA approach. RSSA is a unique and efficient sampling technique that aims to enhance the accuracy of statistical estimates while reducing the cost and effort of data collection. Unlike conventional random sampling methods, where individual items are randomly selected from a population, RSSA involves selecting entire sets of items and arranging them in order based on a specific criterion. Additional details about the RSSA technique can be explored in references such as^27–30^.

Within the realm of science, RSSA finds numerous applications, particularly within environmental and ecological studies, where the primary emphasis lies in the development of cost-effective and efficient sampling methods. Reference^26^ played a pioneering role in establishing the theoretical framework of RSSA, especially in situations where the quantification of sample items proves overly expensive or unfeasible. In such cases, although the variable to be monitored can be ranked more easily and inexpensively than directly measured, as argued by the authors. The authors asserted that when it comes to estimating the mean of a population, utilizing RSSA holds greater value and is more advantageous compared to employing SRSA. This notion was further supported by the mathematical demonstration presented in Ref.^31^, indicating that the mean estimation through RSSA surpasses the performance of SRSA. Within the realm of reliability analysis, Ref.^32^ delved into the task of estimating the reliability of stress-strength, denoted as $P(Y^{*}<X^{*}<Z^{*})$, applicable to a unit characterized by a strength denoted as $X^{*}$, along with lower bound stress denoted as $Y^{*}$, and upper bound stress denoted as $Z^{*}$ using RSSA technique.

Reference^33^ detailed the Bayesian approach for parameter estimation in PSALTE data, assuming an exponential distribution. The researchers in^34^ studied different point predictors, including the best-unbiased, conditional median, and Bayes point predictors. Their study focused on predicting future order statistics based on PSALTE data that are believed to follow a Rayleigh distribution.

This paper introduces the implementation of CSPALTE through ORSSA. The research is carried out with the underlying assumption that the lifespan of an item under operational stress follows a half-logistic distribution. Utilizing type-I hybrid censoring, Bayesian estimation is investigated, with a particular focus on estimating pertinent parameters. Both SLFU and ASLFU are employed in the investigation. Generally, we are motivated to present this paper for the following reasons:

1. Traditional CSALTE methods often assume complete and precise information about the lifetimes of products, leading to potential biases and inaccurate reliability predictions. The incorporation of ordered ranked set sampling facilitates more precise and efficient parameter estimation by strategically selecting the most informative subsets, thereby reducing estimation errors and enhancing the reliability of the results.
2. The proposed methodology integrates type-I hybrid censoring, accommodating scenarios where the continuous monitoring of product lifetimes is impractical or costly. This feature enables the optimization of resources by combining continuous monitoring with periodic inspections, ensuring a balance between data accuracy and cost-effectiveness in the context of reliability assessments.
3. Real-life reliability (engineering) data often exhibit skewness and non-normality, violating assumptions of classical statistical methods. Bayesian estimation, coupled with ordered ranked set sampling, provides a robust framework capable of handling skewed and non-normally distributed data. This allows for more accurate modeling of the underlying reliability distributions, particularly in situations where traditional methods may falter.
4. The proposed methodology is designed to be versatile and applicable across diverse industries where ALTE is crucial for ensuring the longevity and reliability of products. Industries such as electronics, automotive, and aerospace can benefit from this research by obtaining more accurate estimates of product lifetimes, ultimately leading to improved decision-making processes and enhanced product quality.
5. This work contributes to the advancement of Bayesian statistical techniques in the realm of reliability engineering. By combining ORSSA with Bayesian estimation under type-I hybrid censoring, we aim to push the boundaries of current methodologies, opening avenues for further exploration and refinement of Bayesian approaches in reliability analysis.
6. Applying the Bayesian estimation to provide a natural way to quantify and propagate uncertainty through the analysis, which is essential in reliability engineering where uncertainty is inherent.
7. That being said, the proposed Bayesian estimation framework for CSPALTE based on ORSSA under type-I hybrid censoring addresses key challenges in reliability engineering, offering a methodological leap towards more accurate, resource-efficient, and robust parameter estimation in the assessment of product lifetimes.

The following portions of the paper are arranged as follows: Under the CSPALTE, the ORSSA is described in section “Model description”. In sections “Bayes estimation based on ORSSA under CSPALTE” and “Bayes estimation based on a SRSA under CSPALTE”, respectively, Bayesian estimation under type-I hybrid censoring using ORSSA and SRSA is investigated. Section “Real data set: Light-Emitting Diodes” provides a real-world example. Sections “Simulation study” and “Conclusions, discussion, and some potential future points” contain the simulation studies and findings, respectively.

## Model description

In the realm of reliability testing, the CSPALTE method, as detailed in^14^ and^35^, adheres strictly to either utilization or accelerated conditions for every tested item. A pivotal player in this methodology is the tampered RV (TRV) model, extensively explained in^36^. This model finds its application within the CSPALTE technique.

The crux of CSPALTE lies in the segregation of total test items into two distinct groups. The first group undergoes testing under standard stress denoted as $v_{0}$, while the second group is subjected to accelerated stress denoted as $v_{1}$. It’s a meticulous approach, strategically dividing the items to gather insights under different stress conditions.

As per the CSPALTE technique, let’s delve into the specifics. Consider $m_{1}$ as the count of items from group 1 that fail under stress $v_{0}$ with their lifetime RV denoted as $Z=T$. The method operates under the assumption that the CDFU and PDFU of an item’s lifetime *Z* in group 1 are defined by (1) and (2), respectively (with a simple substitution of *t* by *z*). This nuanced approach provides a comprehensive understanding of the lifetime characteristics of the tested items, paving the way for robust reliability analysis.

Furthermore, consider the quantity $m_{2}$ as the count of items within group 2 that undergo failure when exposed to stress $v_{1}$. The TRV model, a key player in this analysis, dictates that the lifetime *Z* of an item experiencing stress $v_{1}$ can be derived by dividing its lifetime under standard stress $v_{0}$ by an acceleration factor $\mu$ greater than 1 (expressed as $Z=T/\mu$). This relationship highlights the role of acceleration in influencing the lifespan of the tested items.

Expanding upon this concept, the CSPALTE technique leverages the TRV model to unravel the statistical characteristics of items in group 2. Drawing from the established CDFU under utilization conditions, referred to as CDFU (1), and the PDFU under utilization conditions, referred to as PDFU (2) for the lifetime variable *T*, we can now articulate the subsequent expressions for the CDFU and PDFU of an item’s lifetime variable *Z* in group 2. These expressions serve as a vital lens through which we can dissect and understand the distribution patterns of lifetimes for items undergoing accelerated stress conditions, offering a nuanced perspective in the realm of reliability analysis, where

3 $$\begin{aligned}{} & {} H_{2}(z)=\displaystyle \frac{1- e^{-\mu z/\eta }}{1+ e^{-\mu z/\eta }}| z>0,\; (\;\eta>0,\; \mu >1), \end{aligned}$$

4 $$\begin{aligned}{} & {} h_{2}(z)=\displaystyle \frac{2\mu e^{-\mu z/\eta }}{\eta \left( 1+ e^{-\mu z/\eta }\right) ^{2}}| z>0,\; (\eta>0,\; \mu >1). \end{aligned}$$

CDFU (1) (after replacing *t* by *z*) and CDFU (3) can be merged in a single equation as follows:

For $$O=1,2$$,

5 $$\begin{aligned} H_{O}(z)=\displaystyle \frac{1- e^{-\mu ^{O-1} z/\eta }}{1+ e^{-\mu ^{O-1} z/{\eta }}}| z>0,\; (\eta>0,\; \mu >1). \end{aligned}$$

Similarly, for PDFUs (2) and (4),

6 $$\begin{aligned} h_{O}(z)=\displaystyle \frac{2\,\mu ^{O-1} e^{-\mu ^{O-1} z/\eta }}{\eta \left( 1+ e^{-\mu ^{O-1} z/\eta }\right) ^{2}}| z>0, \; (\eta>0,\; \mu >1). \end{aligned}$$

### RSSA under CSPALTE

**Figure 1:**
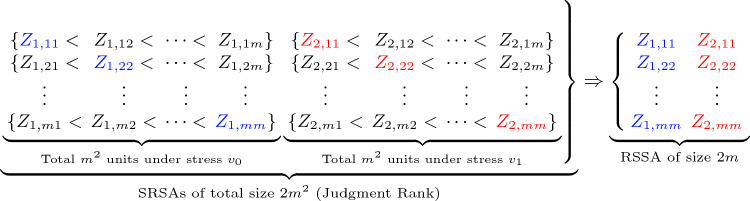
A ranked set sampling procedure under CSPALTE.

Under CSPALTE, the following appro ach can be considered to obtain a RSSA of size 2*m*:

1. Assign a fixed value for *m*.
2. Begin by selecting a total of $2 m^{2}$ items from a specific population, and then meticulously organize them into 2*m* SRSAs. Ensuring uniformity, each of these SRSAs is designed to have an identical size, denoted by *m*, see Fig. 1. This systematic approach to sampling guarantees a comprehensive representation of the population, as the selected items are distributed evenly across the multiple SRSAs. The uniform size of each sample aids in maintaining statistical integrity, facilitating a thorough and unbiased exploration of the chosen subset. This strategic division sets the stage for a meticulous analysis of the population’s characteristics, allowing for meaningful insights to be gleaned from the sampled items.
3. Set $$r=1$$.
4. In the systematic implementation of CSPALTE, the initial step involves a meticulous division of the items earmarked for examination into two distinct groups. Each of these groups is meticulously constructed to represent a SRSA, comprising precisely *m* items. This strategic sampling ensures a representative subset of the population under scrutiny, setting the stage for a comprehensive analysis of the items’ performance under varying stress conditions. Within the CSPALTE framework, a pivotal facet of the experimental design involves subjecting these two groups of items to distinct stress environments. The first group undergoes scrutiny and evaluation under what is referred to as the standard stress, denoted as $v_0$. This standard stress level serves as a control or baseline condition against which subsequent observations and results can be compared. It provides a reference point for gauging the inherent reliability and longevity of the items when subjected to a stress level commonly encountered in their intended operational environment. Simultaneously, the second group undergoes a more rigorous examination under an accelerated stress denoted as $v_1$. This accelerated stress level is deliberately chosen to exert higher demands on the items, simulating conditions that expedite the aging or wear-and-tear processes. By subjecting this group to an elevated stress environment, the CSPALTE methodology aims to accelerate the occurrence of failures and gather valuable data on the items’ response to heightened operational stresses.
5. Order the SRSAs in each group without practical measurement.
6. In the *O*-th ordered SRSA, $O=1, 2$, a single item is measured.
7. In group *O*, the *r*-th smallest item, say $Z_{O, rr}, O=1,2$, is measured.
8. Set $r=r+1$. If $r=m+1$, then halt the previous steps and proceed to Step 10. If not, the smallest item in group *O*, say $Z_{O, r+1 r+1}, O=1, 2$, is measured.
9. Iterate Steps 4–8.
10. A RSSA of size 2*m* is now generated under CSPALTE, see Fig. 1.
11. The one-cycle RSSA of size 2*m*
  $$\begin{aligned} {\textbf {Z}}_{RSSA}= \{ Z_{1, 11}, Z_{1, 22}, \dots, Z_{1, mm}, \, Z_{2, 11}, Z_{2, 22}, \dots, Z_{2, mm}\} \end{aligned}$$
  not only streamlines the data collection process but also enhances the statistical efficiency of the estimation procedures, ensuring that the information gleaned from the experiment is both robust and insightful. To provide a clearer understanding, let’s delve into the specifics of the notation. Consider the notation $Z_{2,44}$, where it signifies the fourth smallest item within the fourth sample belonging to the second group. This notation is instrumental in precisely identifying and referencing specific elements within the structured sampling framework. In essence, it efficiently communicates the sample index, group affiliation, and the position of the item within that particular sample.
12. Iterate Steps 2–9 ${\mathcal {U}}$ cycles to obtain a RSSA of size $2m {\mathcal {U}}$. The obtained data are shown by
  $$\begin{aligned} \begin{aligned} {\textbf {Z}}_{({\mathcal {U}}){RSSA}}=&\{Z_{1,1, 11}, Z_{1,1, 22}, \dots, Z_{1,1, mm}, \, Z_{1,2, 11}, Z_{1,2, 22}, \dots, Z_{1,2, mm},\\&\,\, Z_{2,1, 11}, Z_{2,1, 22}, \dots, Z_{2,1, mm}, \, Z_{2,2, 11}, Z_{2,2, 22}, \dots, Z_{2,2, mm},\\&\qquad \qquad \qquad \qquad \qquad \quad \vdots \qquad \qquad \qquad \qquad \quad \\&Z_{{\mathcal {U}},1, 11}, Z_{{\mathcal {U}},1, 22}, \dots, Z_{{\mathcal {U}},1, mm}, \, Z_{{\mathcal {U}},2, 11}, Z_{{\mathcal {U}},2, 22}, \dots, Z_{{\mathcal {U}},2, mm}\}. \end{aligned} \end{aligned}$$

Now, suppose that ${\textbf {Z}}_{RSSA}$ is a 1-cycle RSSA from a population under CSPALTE with CDFU (5) and PDFU (6). Then, the CDFU and PDFU of $Z_{O, kk}, i=1, 2$, indicated by $H_{O, k:m}$ and $h_{O, k:m}$, are in fact the CDFU and PDFU of the *k*-th order statistic in group *i*. So, they can be expressed as follows, see^37^ and^38^,

7 $$\begin{aligned}{} & {} \begin{aligned} H_{O, k:m}(z)=\sum _{w=k}^{m} \left( {\begin{array}{c}m\\ w\end{array}}\right) [H_{O}(z)]^{w} [1-H_{O}(z)]^{m-w}, \end{aligned} \end{aligned}$$

8 $$\begin{aligned}{} & {} \begin{aligned} h_{O, k:m}(z)=k \left( {\begin{array}{c}m\\ k\end{array}}\right) [H_{O}(z)]^{k-1} [1-H_{O}(z)]^{m-k} h_{O}(z), \end{aligned} \end{aligned}$$

where $H_{O}(z)$ and $h_{O}(z)$ are given by (5) and (6), respectively.

CDFU (7) and PDFU (8) can be rewritten as

9 $$\begin{aligned}{} & {} \begin{aligned} H_{O, k:m}(z)=1-\sum _{w=1}^{k} d^{*}_{w,k}(m) [1-H_{O}(z)]^{m+w-k}, \end{aligned} \end{aligned}$$

10 $$\begin{aligned}{} & {} \begin{aligned} h_{O, k:m}(z)=\sum _{w=0}^{k-1} d_{w,k}(m) [1-H_{O}(z)]^{m+w-k} h_{O}(z), \end{aligned} \end{aligned}$$

where

11 $$\begin{aligned} \left. \begin{aligned} d^{*}_{w,k}(m)&=\displaystyle \frac{d_{w-1,k}(m)}{m+w-k},\\ d_{w,k}(m)&=(-1)^{w} k \left( {\begin{array}{c}k-1\\ w\end{array}}\right) \left( {\begin{array}{c}m\\ k\end{array}}\right) . \end{aligned}\right\} \end{aligned}$$

### RSSA from CSPALTE under type-I hybrid censoring

In the context of CSPALTE, the acquisition of the type-I hybrid censored ordered 1-cycle RSSA unfolds through the following procedure: For each *O* in the range of 1–2, assume that the experimental time $\tau _{O}$ and the count of observed failures *s* are predetermined before the commencement of the experiment. Let it be known that the lifetimes of *m* units designated for testing (where *m* exceeds another pre-assigned value *s*) follow independent RVs with non-identically distributed (IRVNID) CDFU $H_{O, k:m}(z)$ [see Eq. (9)] and PDFU $h_{O, k:m}(z)$ [see Eq. (10)]. The experimenter then makes a decisive determination to conclude the experiment at either the occurrence of the *s*-th failure or at time $\tau _{O}$, depending on which event transpires first.

If the *s*-th failure transpires prior to the designated time $\tau _{O}$, the protocol dictates the removal of all the remaining surviving units, denoted as $m-s$, from the test. This action effectively brings the test to a conclusion at the point of the *s*-th failure time.

Conversely, if the *s*-th failure does not manifest before the specified time $\tau _{O}$ and only $b_{O}$ failures, where $b_{O}$ is less than or equal to *s*, occur before $\tau _{O}$, then, at the precise moment $\tau _{O}$, the remaining surviving units, totaling $m-b_{O}$, are removed from the test. This outcome leads to the termination of the test at $\tau _{O}$.

Now, suppose that the observations given in Step 11, Section “RSSA under CSPALTE”, have been ordered such that $y_{O, j}\equiv z_{O,jj:mm}$. Then type-I hybrid censoring gives rise to the following two observational cases:

- *Case 1* $$y_{O, 1}\le \dots \le y_{O, s}$$, if $$y_{O, s} \le \tau _{O}$$.
- *Case 2* $$y_{O, 1}\le \dots \le y_{O, b_{O}}\le \tau _{O}< y_{ b_{O}+1}$$,    $$b_{O} < s$$ if $$\tau _{O}< y_{O, s}$$.

Following this process, the resultant data set derived from the described operation is termed as a type-I hybrid 1-cycle ORSSA. It is specifically designated by:

12 $$\begin{aligned} {\textbf {y}}=\{\{y_{1, 1} \le y_{1, 2}\le \dots \le y_{1, c_{O}}\}, \{y_{2, 1}\le y_{2, 2} \le \dots \le y_{2, c_{O}}\}\}, \end{aligned}$$

where

$$\begin{aligned} c_{O}=\left\{ \begin{array}{ll} s, &{} \quad \text {if}\quad y_{O, s} \le \tau _{O},\\ b_{O}, &{} \quad \text {if}\quad y_{O, s} > \tau _{O}. \end{array}\right. \end{aligned}$$

## Bayes estimation based on ORSSA under CSPALTE

Based on the insights provided by Balakrishnan^39^ and considering the 1-cycle ORSSA (12), the formulation of the likelihood function (LHF) under the conditions of type-I hybrid censoring can be articulated as follows:

13 $$\begin{aligned} \begin{aligned} \mathbb {L}(\eta ,\mu ;{\textbf {y}})&\varpropto \prod _{O=1}^{2} \left[ \sum _{ P[O]} \prod _{K=1}^{c_{O}} h_{O, r_{O,k}}(y_{O,k}) \prod _{k=c_{O}+1}^{m} [1-H_{O, r_{O,k}}(y_{O}^{*})]\right] , \end{aligned} \end{aligned}$$

where $\sum _{ P[O]}$ indicates the summation over all *m*! permutations $(r_{O, 1}, r_{O, 2},\ldots , r_{O, m})$ of $(1, 2,\ldots , m)$, $\text {{y}} =(\text {{y}}_{1}, \text {{y}}_{2})$, $\text {{y}}_{O} =(y_{O, 1}, \dots , y_{O, c_{O}}),\, O=1, 2$ and

14 $$\begin{aligned} y_{O}^{*}=\left\{ \begin{aligned}&y_{O,s}, \quad \quad \text {if}\quad y_{O, s} \le \tau _{O},\\&\tau _{O}, \quad \quad \,\,\, \text {if}\quad y_{O, s} > \tau _{O}. \end{aligned}\right. \end{aligned}$$

From (13), the LHF can be written as follows:

15 $$\begin{aligned} \begin{aligned} \mathbb {L}(\eta ,\mu ;\text {{y}})&\varpropto \prod _{O=1}^{2}\text {Per} \,{{\varvec{\Theta }}_{\textbf {O}}}, \end{aligned} \end{aligned}$$

where $\text {Per}\, {{\varvec{\Theta }}_{\textbf {O}}}=\sum _{ P[O]} \prod _{K=1}^{m} e_{k,r_{O, k}}$ indicates the permanent of a square real matrix ${{\varvec{\Theta }}_{\textbf {O}}}=(e_{O, k})$ of size $m \times m$ ,

$$\begin{aligned}{{\varvec{\Theta }}_{\textbf {O}}}= \left( \begin{array}{ccccc} h_{O, 1}(y_{O, 1}) &{} \quad h_{O, 2}(y_{O, 1}) &{} \dots &{} \quad h_{O, m}(y_{O, 1}) &{} \quad \\ \vdots &{} \quad \vdots &{} \quad \ddots &{} \quad \vdots &{} \quad \\ h_{O, 1}(y_{O, c_{O}}) &{} \quad h_{O, 2}(y_{O, c_{O}}) &{} \quad \dots &{} \quad h_{O, m}(y_{O, c_{O}}) &{} \quad \\ 1-H_{j, 1}(y_{O}^{*}) &{} \quad 1-H_{j, 2}(y_{O}^{*}) &{} \quad \dots &{} \quad 1-H_{j, n}(y_{O}^{*}) &{} \quad \end{array}\right) _{\big \} (m-c_{O}) \quad \text {rows}.} \end{aligned}$$

By substituting CDFU (9) and PDFU (10) in (13), the LHF can take the next form

16 $$\begin{aligned} \begin{aligned} \mathbb {L}(\eta ,\mu ;\text {{y}})&\varpropto \prod _{O=1}^{2} \left[ \sum _{ P[O]} \left( \prod _{K=1}^{c_{O}} \sum _{w=0}^{r_{O,k}-1} d_{w,r_{O,k}}(m) [1-H_{O}(y_{O,k})]^{m+w-r_{O,k}} h_{O}(y_{O,k}) \right. \right. \\&\quad \left. \left. \times \prod _{k=c_{O}+1}^{m} \sum _{w=1}^{r_{O,k}} d^{*}_{w,r_{O,k}}(m) [1-H_{O}(y_{O}^{*})]^{m+w-r_{O,k}} \right) \right] .\\ \end{aligned} \end{aligned}$$

Based on the CDFU (5) and the PDFU (6) and using the next relations

17 $$\begin{aligned} \left. \begin{aligned} \prod _{K=1}^{c_{O}} \sum _{w=0}^{r_{O,k}-1} \varvec{\Psi }_{w}(r_{O,k})&=\sum _{\gamma _{O,1}=0}^{r_{O,1}-1}\sum _{\gamma _{O,2}=0}^{r_{O,2}-1}\dots \sum _{\gamma _{O,c_{O}}=0}^{r_{O,c_{O}}-1} \prod _{K=1}^{c_{O}} \varvec{\Psi }_{\gamma _{O,k}}(r_{O,k}),\\ \prod _{k=c_{O}+1}^{m} \sum _{w=1}^{r_{O,k}} \varvec{\Psi }^{*}_{w}(r_{O,k})&= \sum _{\varphi _{O,c_{O}+1}=1}^{r_{O,c_{O}+1}}\sum _{\varphi _{O,c_{O}+2}=1}^{r_{O,c_{O}+2}}\dots \sum _{\varphi _{O,m}=1}^{r_{O,m}} \prod _{k=c_{O}+1}^{m} \varvec{\Psi }^{*}_{\varphi _{O,k}}(r_{O,k}),\\ \end{aligned}\right\} \end{aligned}$$

the LHF can be expressed as

18 $$\begin{aligned} \begin{aligned} \mathbb {L}(\eta ,\mu ;\text {{y}})&\varpropto \prod _{O=1}^{2} \left[ \sum _{ P[O]}\sum _{\varvec{\gamma }_{O},\varvec{\varphi }_{O}}^{c_{O},m} \left( \Upsilon _{\varvec{\gamma }_{O},\varvec{\varphi }_{O}}(\varvec{ r}_{O})\,\, B_{\varvec{\gamma }_{O}}( {\textbf {y}}_{O})\,\, D_{\varvec{\varphi }_{O}}( {\textbf {y}}_{O}^{*})\,\, \exp \left[ -\frac{\mu ^{0-1}}{\eta } \Delta _{\varvec{\gamma }_{O},\varvec{\varphi }_{O}}( {\textbf {y}}_{O})\right] \right) \right] , \end{aligned} \end{aligned}$$

where $$\varvec{r}_{O} =(r_{O, 1}, \dots , r_{O, c_{O}}, r_{O, c_{O}+1}, \dots , r_{O, m})$$, $$\varvec{\gamma }_{O} =(\gamma _{O, 1}, \dots , \gamma _{O, c_{O}})$$, $$\varvec{\varphi }_{O} =(\varphi _{O, c_{O}+1}, \dots , \varphi _{O, m})$$, $$O=1, 2$$, and

19 $$\begin{aligned}{} & {} \begin{aligned} \sum _{\varvec{\gamma }_{O},\varvec{\varphi }_{O}}^{c_{O},m}=\sum _{\gamma _{O,1}=0}^{r_{O,1}-1}\sum _{\gamma _{O,2}=0}^{r_{O,2}-1}\dots \sum _{\gamma _{O,c_{O}}=0}^{r_{O,c_{O}}-1}. \sum _{\varphi _{O,c_{O}+1}=1}^{r_{O,c_{O}+1}}\sum _{\varphi _{O,c_{O}+2}=1}^{r_{O,c_{O}+2}}\dots \sum _{\varphi _{O,m}=1}^{r_{O,m}}, \end{aligned} \end{aligned}$$

20 $$\begin{aligned}{} & {} \begin{aligned} \Upsilon _{\varvec{\gamma }_{O},\varvec{\varphi }_{O}}(\varvec{ r}_{O} )=\left[ \prod _{K=1}^{c_{O}} d_{\gamma _{O,k},r_{O,k}}(m)\right] \left[ \prod _{k=c_{O}+1}^{m} d^{*}_{\varphi _{O,k},r_{O,k}}(m)\right] , \end{aligned} \end{aligned}$$

21 $$\begin{aligned}{} & {} \begin{aligned} B_{\varvec{\gamma }_{O}}( {\textbf {y}}_{O})=\prod _{K=1}^{c_{O}} \frac{\mu ^{0-1}}{2 \eta } \left( \frac{1}{2}\left\{ 1+\text{ exp }\left[ -\frac{\mu ^{0-1}}{\eta } y_{O,k} \right] \right\} \right) ^{-m-\gamma _{O,k}+r_{O,k}-2}, \end{aligned} \end{aligned}$$

22 $$\begin{aligned}{} & {} \begin{aligned} D_{\varvec{\varphi }_{O}}( {\textbf {y}}_{O}^{*})= \left( \frac{1}{2}\left\{ 1+\text {exp}\left[ -\frac{\mu ^{0-1}}{\eta } y_{O}^{*} \right] \right\} \right) ^{\sum _{k=c_{O}+1}^{m} -m-\varphi _{O,k}+r_{O,k}}, \end{aligned} \end{aligned}$$

23 $$\begin{aligned}{} & {} \begin{aligned} \Delta _{\varvec{\gamma }_{O},\varvec{\varphi }_{O}}( {\textbf {y}}_{O})=\left[ \sum _{K=1}^{c_{O}} (m+\gamma _{O,k}-r_{O,k}+1)y_{O,k}\right] +\left[ \sum _{k=c_{O}+1}^{m} (m+\varphi _{O,k}-r_{O,k})y_{O}^{*}\right] . \end{aligned} \end{aligned}$$

Due to (17), the LHF can be expressed as

24 $$\begin{aligned} \begin{aligned} \mathbb {L}(\eta ,\mu ;\text {{y}})&\varpropto \sum _{{\varvec{P}}^{*}, \varvec{\gamma }^{*}, \varvec{\varphi }^{*}}^{2, c_{O}, m} \left( \Bigg [ \prod _{O=1}^{2} \Upsilon _{\varvec{\gamma }_{O},\varvec{\varphi }_{O}}(\varvec{ r}_{O})\,\, B_{\varvec{\gamma }_{O}}( {\textbf {y}}_{O})\,\, D_{\varvec{\varphi }_{O}}( {\textbf {y}}_{O}^{*})\,\,\Bigg ] \exp \left[ -\sum _{O=1}^{2} \frac{\mu ^{O-1}}{\eta } \Delta _{\varvec{\gamma }_{O},\varvec{\varphi }_{O}}( {\textbf {y}}_{O})\right] \right) , \end{aligned} \end{aligned}$$

where $$\varvec{P}^{*}=( P[1], P[2])$$, $$\varvec{\gamma }^{*} =(\varvec{\gamma }_{1}, \varvec{\gamma }_{2})$$, $$\varvec{\gamma }_{O} =(\gamma _{O, 1}, \dots , \gamma _{O, c_{O}})$$, $$\varvec{\varphi }^{*} =(\varvec{\varphi }_{1} , \varvec{\varphi }_{2})$$,

$$\varvec{\varphi }_{O} =(\varphi _{O, c_{O}+1}, \dots , \varphi _{O, m})$$, $$O=1, 2$$, and

25 $$\begin{aligned} \begin{aligned} \sum _{{\varvec{P}}^{*}, \varvec{\gamma }^{*}, \varvec{\varphi }^{*}}^{2, c_{O}, m}= \prod _{O=1}^{2} \sum _{ P[O]}\sum _{\varvec{\gamma }_{O},\varvec{\varphi }_{O}}^{c_{O},m}= \sum _{ P[1]}\sum _{\varvec{\gamma }_{1},\varvec{\varphi }_{1}}^{c_{1},m} \sum _{ P[2]}\sum _{\varvec{\gamma }_{2},\varvec{\varphi }_{2}}^{c_{2},m}, \end{aligned} \end{aligned}$$

where $\sum _{\varvec{\gamma }_{O},\varvec{\varphi }_{O}}^{c_{O},m}$ is given by (19).

For ${\mathcal {U}}$-cycle ORSSA, the LHF under type-I hybrid censoring is then given by

26 $$\begin{aligned} \begin{aligned} \mathbb {L}(\eta ,\mu ;\text {{y}})&\varpropto \prod _{\rho =1}^{{\mathcal {U}}}\left[ \sum _{{\varvec{P}}^{*}_{\rho }, \varvec{\gamma }^{*}_{\rho }, \varvec{\varphi }^{*}_{\rho }}^{2, c_{\rho , O}, m} \Bigg ( \Bigg [ \prod _{O=1}^{2} \Upsilon _{\varvec{\gamma }_{\rho , O},\varvec{\varphi }_{\rho , O}}(\varvec{ r}_{\rho , O})\,\, B_{\varvec{\gamma }_{\rho , O}}( {\textbf {y}}_{\rho , O})\,\, D_{\varvec{\varphi }_{\rho , O}}( {\textbf {y}}_{\rho , O}^{*})\,\,\Bigg ] \right. \\&\quad \times \left. \left. \exp \left[ -\sum _{O=1}^{2} \frac{\mu ^{0-1}}{\eta } \Delta _{\varvec{\gamma }_{\rho , O},\varvec{\varphi }_{\rho , O}}( {\textbf {y}}_{\rho , O})\right] \right) \right] . \end{aligned} \end{aligned}$$

Using the relations given in (17), the LHF can be rewritten as

27 $$\begin{aligned} \begin{aligned} \mathbb {L}(\eta ,\mu ;\text {{y}})&\varpropto \sum _{{\varvec{P}}^{**}, \varvec{\gamma }^{**}, \varvec{\varphi }^{**}}^{2, c_{\rho , O}, m} \left( \left[ \prod _{\rho =1}^{{\mathcal {U}}} \prod _{O=1}^{2} \Upsilon _{\varvec{\gamma }_{\rho , O},\varvec{\varphi }_{\rho , O}}(\varvec{ r}_{\rho , O})\,\, B_{\varvec{\gamma }_{\rho , O}}( {\textbf {y}}_{\rho , O})\,\, D_{\varvec{\varphi }_{\rho , O}}( {\textbf {y}}_{\rho , O}^{*})\,\,\right] \right. \\&\quad \times \left. \exp \left[ - \sum _{\rho =1}^{{\mathcal {U}}}\sum _{O=1}^{2} \frac{\mu ^{0-1}}{\eta } \Delta _{\varvec{\gamma }_{\rho , O},\varvec{\varphi }_{\rho , O}}( {\textbf {y}}_{\rho , O})\right] \right) , \end{aligned} \end{aligned}$$

where

28 $$\begin{aligned}{} & {} \begin{aligned} \sum _{{\varvec{P}}^{**}, \varvec{\gamma }^{**}, \varvec{\varphi }^{**}}^{2, c_{\rho , O}, m}=\prod _{\rho =1}^{{\mathcal {U}}} \sum _{{\varvec{P}}^{*}_{\rho }, \varvec{\gamma }^{*}_{\rho }, \varvec{\varphi }^{*}_{\rho }}^{2, c_{\rho , O}, m}= \sum _{{\varvec{P}}^{*}_{1}, \varvec{\gamma }^{*}_{1}, \varvec{\varphi }^{*}_{1}}^{2, c_{\rho , O}, m}\dots \sum _{{\varvec{P}}^{*}_{{\mathcal {U}}}, \varvec{\gamma }^{*}_{{\mathcal {U}}}, \varvec{\varphi }^{*}_{{\mathcal {U}}}}^{2,c_{\rho , O}, m}, \end{aligned} \end{aligned}$$

29 $$\begin{aligned}{} & {} \begin{aligned} \sum _{{\varvec{P}}^{*}_{\rho }, \varvec{\gamma }^{*}_{\rho }, \varvec{\varphi }^{*}_{\rho }}^{2, c_{\rho , O}, m}= \sum _{ P[\rho , 1]}\sum _{\varvec{\gamma }_{\rho , 1},\varvec{\varphi }_{\rho , 1}}^{c_{\rho , O}, m}. \sum _{ P[\rho , 2]} \sum _{\varvec{\gamma }_{\rho , 2},\varvec{\varphi }_{\rho , 2}}^{c_{\rho , O}, m}, \end{aligned} \end{aligned}$$

30 $$\begin{aligned}{} & {} \begin{aligned} \sum _{\varvec{\gamma }_{\rho , O},\varvec{\varphi }_{\rho , O}}^{c_{\rho , O}, m}=\sum _{\gamma _{\rho , O,1}=0}^{r_{\rho , O,1}-1} \sum _{\gamma _{\rho , O,2}=0}^{r_{\rho , O,2}-1}\dots \sum _{\gamma _{\rho , O, c_{\rho , O}}=0}^{r_{\rho , O,c_{\rho , O}}-1}. \sum _{\varphi _{\rho , O,c_{\rho , O}+1}=1}^{r_{\rho , O,c_{\rho , O}+1}}\sum _{\varphi _{\rho , O,c_{\rho , O}+2}=1}^{r_{\rho , O,c_{\rho , O}+2}}\dots \sum _{\varphi _{\rho , O,m}=1}^{r_{\rho , O,m}}, \end{aligned} \end{aligned}$$

and $$\varvec{r}_{\rho , O} =(r_{\rho , O, 1}, \dots , r_{\rho , O, c_{\rho , O}}, r_{\rho , O, c_{\rho , O}+1}, \dots , r_{\rho , O, m})$$, $$\rho =1, \dots , {\mathcal {U}}$$, $$O=1, 2$$, $$\varvec{P}^{**}=( \varvec{P}_{1}^{*}, \dots , \varvec{P}_{{\mathcal {U}}}^{*})$$, $$\varvec{P}_{\rho }^{*}=( P[\rho , 1], P[\rho , 2])$$, $$\varvec{\gamma }^{**}=( \varvec{\gamma }_{1}^{*}, \dots , \varvec{\gamma }_{{\mathcal {U}}}^{*})$$, $$\varvec{\gamma }_{\rho }^{*} =(\varvec{\gamma }_{\rho , 1}, \varvec{\gamma }_{q, 2})$$, $$\varvec{\gamma }_{\rho , O} =(\gamma _{q, O, 1}, \dots , \gamma _{\rho , O, c_{\rho , O}})$$, $$\varvec{\varphi }^{**}=( \varvec{\varphi }_{1}^{*}, \dots , \varvec{\varphi }_{{\mathcal {U}}}^{*})$$, $$\varvec{\varphi }_{\rho }^{*} =(\varvec{\varphi }_{\rho , 1}, \varvec{\varphi }_{\rho , 2})$$, $$\varvec{\varphi }_{\rho , O} =(\varphi _{\rho , O, c_{\rho , O}+1}, \dots , \varphi _{\rho , O, m})$$.

### Prior and posterior distributions

In Bayesian inference, it is assumed that the unknown parameters are considered as RVs controlled by a collective prior density function. With the use of past data and learned information, this prior density function can be determined. When prior knowledge is limited, Bayesian inference can be applied with non-informative priors. In this context, we consider $\eta$ and $\mu$ are independent variables with the following joint prior density,

31 $$\begin{aligned} \pi (\eta ,\mu )=\pi _{1}(\eta )\, \pi _{2}(\mu ), \end{aligned}$$

$\pi _{1}(\eta )$ has Weibull model with two parameters $b_1$ and $b_2$ as follows:

32 $$\begin{aligned} \pi _{1}(\eta )= b_{1} b_{2}^{-b_{1}} \eta ^{b_{1}-1} \, \exp \left[ {- \left( \frac{\eta }{b_{2}}\right) ^{b_{1}}}\right] | \eta>0,\quad (b_{1}, b_{2})>0, \end{aligned}$$

while $\pi _{2}(\mu )$ is non-informative prior of the following form:

33 $$\begin{aligned} \pi _{2}(\mu )=\frac{1}{\mu }| \mu >1. \end{aligned}$$

Using (32) and (33), joint prior density (31) becomes

34 $$\begin{aligned} \pi (\eta ,\mu )=b_{1} b_{2}^{-b_{1}} \mu ^{-1} \eta ^{b_{1}-1} \, \exp \left[ {- \left( \frac{\eta }{b_{2}}\right) ^{b_{1}}}\right] | \eta>0,\; \mu>1,\quad (b_{1},b_{2})>0. \end{aligned}$$

From (27) and (34), the joint density function of the posterior model of $\eta$ and $\mu$ may be expressed as:

35 $$\begin{aligned} \begin{aligned} \pi ^{*}(\eta ,\mu |\text {{y}})&=\Omega ^{-1}\, \sum _{{\varvec{P}}^{**}, \varvec{\gamma }^{**}, \varvec{\varphi }^{**}}^{2, c_{\rho , O}, m} \Bigg ( \Bigg [ \prod _{\rho =1}^{{\mathcal {U}}} \prod _{O=1}^{2} \Upsilon _{\varvec{\gamma }_{\rho , O},\varvec{\varphi }_{\rho , O}}(\varvec{ r}_{\rho , O})\,\, B_{\varvec{\gamma }_{\rho , O}}( {\textbf {y}}_{\rho , O})\,\, D_{\varvec{\varphi }_{\rho , O}}( {\textbf {y}}_{\rho , O}^{*})\,\,\Bigg ]\\&\quad \times \left. \, \mu ^{-1} \eta ^{b_{1}-1} \,\exp \left[ - \left( \frac{\eta }{b_{2}}\right) ^{b_{1}} - \sum _{\rho =1}^{{\mathcal {U}}}\sum _{O=1}^{2} \frac{\mu ^{0-1}}{\eta } \Delta _{\varvec{\gamma }_{\rho , O},\varvec{\varphi }_{\rho , O}}( {\textbf {y}}_{\rho , O})\right] \right) , \end{aligned} \end{aligned}$$

where

$$\begin{aligned} \begin{aligned} \Omega&= \sum _{{\varvec{P}}^{**}, \varvec{\gamma }^{**}, \varvec{\varphi }^{**}}^{2, c_{\rho , O}, m} \left( \int _{1}^{\infty }\int _{0}^{\infty } \left[ \prod _{\rho =1}^{{\mathcal {U}}} \prod _{O=1}^{2} \Upsilon _{\varvec{\gamma }_{\rho , O},\varvec{\varphi }_{\rho , O}}(\varvec{ r}_{\rho , O})\,\, B_{\varvec{\gamma }_{\rho , O}}( {\textbf {y}}_{\rho , O})\,\, D_{\varvec{\varphi }_{\rho , O}}( {\textbf {y}}_{\rho , O}^{*})\,\,\right] \right. \\&\quad \left. \times \, \mu ^{-1} \eta ^{b_{1}-1} \,\exp \left[ - \left( \frac{\eta }{b_{2}}\right) ^{b_{1}} - \sum _{\rho =1}^{{\mathcal {U}}}\sum _{O=1}^{2} \frac{\mu ^{0-1}}{\eta } \Delta _{\varvec{\gamma }_{\rho , O},\varvec{\varphi }_{\rho , O}}( {\textbf {y}}_{\rho , O})\right] \right) \,\text {d} \eta \,\,\text {d} \mu . \end{aligned} \end{aligned}$$

### Bayesian estimation under asymmetric and symmetric loss functions

Bayesian estimation involves a statistical approach where uncertainty is captured by using probability distributions. The choice of a certain loss-function (LFU) plays a crucial role in this process. The LFU quantifies the discrepancy between predicted values and actual outcomes. In the context of Bayesian estimation, both SLFU and ASLFU are utilized.

SLFUs treat overestimation and underestimation equally. They aim to minimize the overall error between predictions and true values, regardless of the direction of the error.

On the other hand, ASLFUs assign different penalties to overestimation and underestimation. This reflects situations where the cost of making one type of error is higher than the other. Asymmetric losses are especially useful when dealing with skewed or heavy-tailed data.

The selection of a suitable LFU depends on the specific problem and the associated costs of prediction errors. Bayesian estimation allows incorporating prior information, uncertainty, and the chosen LFU to make more informed and context-aware decisions.

The superiority of estimators for model parameters is evident when employing ASLFUs compared to estimators obtained using SLFUs. Numerous authors have explored the Bayesian estimation of underlying parameters using both SLFU and ASLFU, as evidenced in various works, including references such as^40^–^43^.

The Bayes estimates (BESs) of $\eta$ and $\mu$, due to SLFU and ASLFU are

1. The squared error LFU (SELFU) is defined as follows:
  $$\begin{aligned} {\mathcal {L}}({\hat{\Lambda }},\Lambda )\propto \left( {{\hat{\Lambda }}}-{\Lambda }\right) ^{2}, \end{aligned}$$
  where ${\hat{\Lambda }}$ is the estimation of the parameter $\Lambda$. The BESs of $\Lambda$, based on the SELFU, is given by
  36 $$\begin{aligned} \begin{aligned} {\hat{\Lambda }}_{S}=\text {E}[\Lambda |\text {{y}}]. \end{aligned} \end{aligned}$$
  From (35) and (36) the BESs of $\eta$ and $\mu$, based on the SELFU, are then given, respectively, by
  37 $$\begin{aligned}{} & {} \begin{aligned} {\hat{\eta }}_{S}&=\Omega ^{-1} \sum _{{\varvec{P}}^{**}, \varvec{\gamma }^{**}, \varvec{\varphi }^{**}}^{2, c_{\rho , O}, m} \left( \int _{1}^{\infty }\int _{0}^{\infty } \left[ \prod _{\rho =1}^{{\mathcal {U}}} \prod _{O=1}^{2} \Upsilon _{\varvec{\gamma }_{\rho , O},\varvec{\varphi }_{\rho , O}}(\varvec{ r}_{\rho , O})\,\, B_{\varvec{\gamma }_{\rho , O}}( {\textbf {y}}_{\rho , O})\,\, D_{\varvec{\varphi }_{\rho , O}}( {\textbf {y}}_{\rho , O}^{*})\,\,\right] \right. \\&\quad \times \, \left. \mu ^{-1} \eta ^{b_{1}} \,\exp \left[ - \left( \frac{\eta }{b_{2}}\right) ^{b_{1}} - \sum _{\rho =1}^{{\mathcal {U}}}\sum _{O=1}^{2} \frac{\mu ^{O-1}}{\eta } \Delta _{\varvec{\gamma }_{\rho , O},\varvec{\varphi }_{\rho , O}}( {\textbf {y}}_{\rho , O})\right] \right) \,\text {d} \eta \,\,\text {d} \mu , \end{aligned} \end{aligned}$$
  38 $$\begin{aligned}{} & {} \begin{aligned} {\hat{\mu }}_{S}&=\Omega ^{-1} \sum _{{\varvec{P}}^{**}, \varvec{\gamma }^{**}, \varvec{\varphi }^{**}}^{2, c_{\rho , O}, m} \left( \int _{1}^{\infty }\int _{0}^{\infty } \left[ \prod _{\rho =1}^{{\mathcal {U}}} \prod _{O=1}^{2} \Upsilon _{\varvec{\gamma }_{\rho , O},\varvec{\varphi }_{\rho , O}}(\varvec{ r}_{\rho , O})\,\, B_{\varvec{\gamma }_{\rho , O}}( {\textbf {y}}_{\rho , O})\,\, D_{\varvec{\varphi }_{\rho , O}}( {\textbf {y}}_{\rho , O}^{*})\,\,\right] \right. \\&\quad \times \, \left. \eta ^{b_{1}-1} \,\exp \left[ - \left( \frac{\eta }{b_{2}}\right) ^{b_{1}} - \sum _{\rho =1}^{{\mathcal {U}}}\sum _{O=1}^{2} \frac{\mu ^{O-1}}{\eta } \Delta _{\varvec{\gamma }_{\rho , O},\varvec{\varphi }_{\rho , O}}( {\textbf {y}}_{\rho , O})\right] \right) \,\text {d} \eta \,\,\text {d} \mu . \end{aligned} \end{aligned}$$
2. The weighted SELFU (WSELFU) is defined as follows:
  $$\begin{aligned} {\mathcal {L}}({\hat{\Lambda }},\Lambda )\propto \frac{\left( {{\hat{\Lambda }}}-{\Lambda }\right) ^{2}}{\Lambda }. \end{aligned}$$
  The BES of $\Lambda$, based on the WSELFU, is given by
  39 $$\begin{aligned} \begin{aligned} {\hat{\Lambda }}_{WS}=(\text {E}[\Lambda ^{-1}|\text {{y}}])^{-1}. \end{aligned} \end{aligned}$$
  From (35) and (39) the BESs of $\eta$ and $\mu$, based on the WSELFU, are then given, respectively, by
  40 $$\begin{aligned}{} & {} \begin{aligned} {\hat{\eta }}_{WS}&=\left\{ \Omega ^{-1} \sum _{{\varvec{P}}^{**}, \varvec{\gamma }^{**}, \varvec{\varphi }^{**}}^{2, c_{\rho , O}, m} \left( \int _{1}^{\infty }\int _{0}^{\infty } \left[ \prod _{\rho =1}^{{\mathcal {U}}} \prod _{O=1}^{2} \Upsilon _{\varvec{\gamma }_{\rho , O},\varvec{\varphi }_{\rho , O}}(\varvec{ r}_{\rho , O})\,\, B_{\varvec{\gamma }_{\rho , O}}( {\textbf {y}}_{\rho , O})\,\, D_{\varvec{\varphi }_{\rho , O}}( {\textbf {y}}_{\rho , O}^{*})\,\,\right] \right. \right. \\&\quad \times \, \left. \left. \mu ^{-1} \eta ^{b_{1}-2} \,\exp \left[ - \left( \frac{\eta }{b_{2}}\right) ^{b_{1}} - \sum _{\rho =1}^{{\mathcal {U}}}\sum _{O=1}^{2} \frac{\mu ^{O-1}}{\eta } \Delta _{\varvec{\gamma }_{\rho , O},\varvec{\varphi }_{\rho , O}}( {\textbf {y}}_{\rho , O})\right] \right) \,\text {d} \eta \,\,\text {d} \mu \right\} ^{-1}. \end{aligned} \end{aligned}$$
  41 $$\begin{aligned}{} & {} \begin{aligned} {\hat{\mu }}_{WS}&=\left\{ \Omega ^{-1} \sum _{{\varvec{P}}^{**}, \varvec{\gamma }^{**}, \varvec{\varphi }^{**}}^{2, c_{\rho , O}, m} \left( \int _{1}^{\infty }\int _{0}^{\infty } \left[ \prod _{\rho =1}^{{\mathcal {U}}} \prod _{O=1}^{2} \Upsilon _{\varvec{\gamma }_{\rho , O},\varvec{\varphi }_{\rho , O}}(\varvec{ r}_{\rho , O})\,\, B_{\varvec{\gamma }_{\rho , O}}( {\textbf {y}}_{\rho , O})\,\, D_{\varvec{\varphi }_{\rho , O}}( {\textbf {y}}_{\rho , O}^{*})\,\,\right] \right. \right. \\&\quad \times \, \left. \left. \mu ^{-2} \eta ^{b_{1}-1} \,\exp \left[ - \left( \frac{\eta }{b_{2}}\right) ^{b_{1}} - \sum _{\rho =1}^{{\mathcal {U}}}\sum _{O=1}^{2} \frac{\mu ^{O-1}}{\eta } \Delta _{\varvec{\gamma }_{\rho , O},\varvec{\varphi }_{\rho , O}}( {\textbf {y}}_{\rho , O})\right] \right) \,\text {d} \eta \,\,\text {d} \mu \right\} ^{-1}. \end{aligned} \end{aligned}$$
3. The precautionary LFU (PRLFU) is defined as follows:
  $$\begin{aligned} {\mathcal {L}}({\hat{\Lambda }},\Lambda )\propto \frac{\left( {{\hat{\Lambda }}}-{\Lambda }\right) ^{2}}{{\hat{\Lambda }}}. \end{aligned}$$
  The BES of $\Lambda$, based on the PRLFU, is given by
  42 $$\begin{aligned} \begin{aligned} {\hat{\Lambda }}_{P}=(\text {E}[\Lambda ^{2}|\text {{y}}])^{\frac{1}{2}}. \end{aligned} \end{aligned}$$
  From (35) and (42) the BESs of $\eta$ and $\mu$, based on the PRLFU, are then given, respectively, by
  43 $$\begin{aligned}{} & {} \begin{aligned} {\hat{\eta }}_{P}=&\left\{ \Omega ^{-1} \sum _{{\varvec{P}}^{**}, \varvec{\gamma }^{**}, \varvec{\varphi }^{**}}^{2, c_{\rho , O}, m} \left( \int _{1}^{\infty }\int _{0}^{\infty } \left[ \prod _{\rho =1}^{{\mathcal {U}}} \prod _{O=1}^{2} \Upsilon _{\varvec{\gamma }_{\rho , O},\varvec{\varphi }_{\rho , O}}(\varvec{ r}_{\rho , O})\,\, B_{\varvec{\gamma }_{\rho , O}}( {\textbf {y}}_{\rho , O})\,\, D_{\varvec{\varphi }_{\rho , O}}( {\textbf {y}}_{\rho , O}^{*})\,\,\right] \right. \right. \\&\quad \times \left. \left. \mu ^{-1} \eta ^{b_{1}+1} \,\exp \left[ - \left( \frac{\eta }{b_{2}}\right) ^{b_{1}} - \sum _{\rho =1}^{{\mathcal {U}}}\sum _{O=1}^{2} \frac{\mu ^{O-1}}{\eta } \Delta _{\varvec{\gamma }_{\rho , O},\varvec{\varphi }_{\rho , O}}( {\textbf {y}}_{\rho , O})\right] \right) \,\text {d} \eta \,\,\text {d} \mu \right\} ^{\frac{1}{2}}. \end{aligned} \end{aligned}$$
  44 $$\begin{aligned}{} & {} \begin{aligned} {\hat{\mu }}_{P}=&\left\{ \Omega ^{-1} \sum _{{\varvec{P}}^{**}, \varvec{\gamma }^{**}, \varvec{\varphi }^{**}}^{2, c_{\rho , O}, m} \left( \int _{1}^{\infty }\int _{0}^{\infty } \left[ \prod _{\rho =1}^{{\mathcal {U}}} \prod _{O=1}^{2} \Upsilon _{\varvec{\gamma }_{\rho , O},\varvec{\varphi }_{\rho , O}}(\varvec{ r}_{\rho , O})\,\, B_{\varvec{\gamma }_{\rho , O}}( {\textbf {y}}_{\rho , O})\,\, D_{\varvec{\varphi }_{\rho , O}}( {\textbf {y}}_{\rho , O}^{*})\,\,\right] \right. \right. \\&\quad \times \, \left. \left. \mu \eta ^{b_{1}-1} \,\exp \left[ - \left( \frac{\eta }{b_{2}}\right) ^{b_{1}} - \sum _{\rho =1}^{{\mathcal {U}}}\sum _{O=1}^{2} \frac{\mu ^{0-1}}{\eta } \Delta _{\varvec{\gamma }_{\rho , O},\varvec{\varphi }_{\rho , O}}( {\textbf {y}}_{\rho , O})\right] \right) \,\text {d} \eta \,\,\text {d} \mu \right\} ^{\frac{1}{2}}. \end{aligned} \end{aligned}$$
4. The logarithmic LFU (LGLFU) is defined as follows:
  $$\begin{aligned} {\mathcal {L}}({\hat{\Lambda }},\Lambda )\propto \left( {\log {\hat{\Lambda }}}-{\log \Lambda }\right) ^{2}. \end{aligned}$$
  The BES of $\Lambda$, based on the LGLFU, is given by
  45 $$\begin{aligned} \begin{aligned} {\hat{\Lambda }}_{LG}=\exp [\text {E}[\log \Lambda |\text {{y}}]]. \end{aligned} \end{aligned}$$
  From (35) and (45) the BESs of $\eta$ and $\mu$, based on the LGLFU, are then given, respectively, by
  46 $$\begin{aligned}{} & {} \begin{aligned} {\hat{\eta }}_{LG}&=\exp \left[ \Omega ^{-1} \sum _{{\varvec{P}}^{**}, \varvec{\gamma }^{**}, \varvec{\varphi }^{**}}^{2, c_{\rho , O}, m} \left( \int _{1}^{\infty }\int _{0}^{\infty } \left[ \prod _{\rho =1}^{{\mathcal {U}}} \prod _{O=1}^{2} \Upsilon _{\varvec{\gamma }_{\rho , O},\varvec{\varphi }_{\rho , O}}(\varvec{ r}_{\rho , O})\,\, B_{\varvec{\gamma }_{\rho , O}}( {\textbf {y}}_{\rho , O})\,\, D_{\varvec{\varphi }_{\rho , O}}( {\textbf {y}}_{\rho , O}^{*})\,\,\right] \right. \right. \\&\quad \times \, \left. \left. \mu ^{-1}\, \log [\eta ]\, \eta ^{b_{1}-1} \,\exp \left[ - \left( \frac{\eta }{b_{2}}\right) ^{b_{1}} - \sum _{\rho =1}^{{\mathcal {U}}}\sum _{O=1}^{2} \frac{\mu ^{0-1}}{\eta } \Delta _{\varvec{\gamma }_{\rho , O},\varvec{\varphi }_{\rho , O}}( {\textbf {y}}_{\rho , O})\right] \right) \,\text {d} \eta \,\,\text {d} \mu \right] . \end{aligned} \end{aligned}$$
  47 $$\begin{aligned}{} & {} \begin{aligned} {\hat{\mu }}_{LG}&=\exp \left[ \Omega ^{-1} \sum _{{\varvec{P}}^{**}, \varvec{\gamma }^{**}, \varvec{\varphi }^{**}}^{2, c_{\rho , O}, m} \left( \int _{1}^{\infty }\int _{0}^{\infty } \left[ \prod _{\rho =1}^{{\mathcal {U}}} \prod _{O=1}^{2} \Upsilon _{\varvec{\gamma }_{\rho , O},\varvec{\varphi }_{\rho , O}}(\varvec{ r}_{\rho , O})\,\, B_{\varvec{\gamma }_{\rho , O}}( {\textbf {y}}_{\rho , O})\,\, D_{\varvec{\varphi }_{\rho , O}}( {\textbf {y}}_{\rho , O}^{*})\,\,\right] \right. \right. \\&\quad \times \left. \left. \, \mu ^{-1} \,\log [\mu ]\, \eta ^{b_{1}-1} \,\exp \left[ - \left( \frac{\eta }{b_{2}}\right) ^{b_{1}} - \sum _{\rho =1}^{{\mathcal {U}}}\sum _{O=1}^{2} \frac{\mu ^{0-1}}{\eta } \Delta _{\varvec{\gamma }_{\rho , O},\varvec{\varphi }_{\rho , O}}( {\textbf {y}}_{\rho , O})\right] \right) \,\text {d} \eta \,\,\text {d} \mu \right] . \end{aligned} \end{aligned}$$
5. The linear exponential LFU (LINEXLFU) is defined as follows:
  $$\begin{aligned} {\mathcal {L}}(\delta )\propto e^{\sigma \zeta }- \sigma \zeta -1| \sigma \ne 0, \end{aligned}$$
  where $\zeta ={\hat{\Lambda }}-\Lambda$. The BES of $\Lambda$, based on the LINEXLFU, is given by
  48 $$\begin{aligned} \begin{aligned} {\hat{\Lambda }}_{LI}=\frac{-1}{\sigma }\log [\text {E}({e^{-\sigma \Lambda }}|\text {{y}})]. \end{aligned} \end{aligned}$$
  From (35) and (48) the BESs of $\eta$ and $\mu$, based on the LINEXLFU, are then given, respectively, by
  49 $$\begin{aligned}{} & {} \begin{aligned} {\hat{\eta }}_{LI}&=\frac{-1}{\sigma }\log \left[ \Omega ^{-1} \sum _{{\varvec{P}}^{**}, \varvec{\gamma }^{**}, \varvec{\varphi }^{**}}^{2, c_{\rho , O}, m} \left( \int _{1}^{\infty }\int _{0}^{\infty } \left[ \prod _{\rho =1}^{{\mathcal {U}}} \prod _{O=1}^{2} \Upsilon _{\varvec{\gamma }_{\rho , O},\varvec{\varphi }_{\rho , O}}(\varvec{ r}_{\rho , O})\,\, B_{\varvec{\gamma }_{\rho , O}}( {\textbf {y}}_{\rho , O})\,\, D_{\varvec{\varphi }_{\rho , O}}( {\textbf {y}}_{\rho , O}^{*})\,\,\right] \right. \right. \\&\quad \times \left. \left. \, \mu ^{-1} \eta ^{b_{1}-1} \,\exp \left[ - \sigma \eta - \left( \frac{\eta }{b_{2}}\right) ^{b_{1}} - \sum _{\rho =1}^{{\mathcal {U}}}\sum _{O=1}^{2} \frac{\mu ^{0-1}}{\eta } \Delta _{\varvec{\gamma }_{\rho , O},\varvec{\varphi }_{\rho , O}}( {\textbf {y}}_{\rho , O})\right] \right) \,\text {d} \eta \,\,\text {d} \mu \right] , \end{aligned} \end{aligned}$$
  50 $$\begin{aligned}{} & {} \begin{aligned} {\hat{\mu }}_{LI}&=\frac{-1}{\sigma }\log \left[ \Omega ^{-1} \sum _{{\varvec{P}}^{**}, \varvec{\gamma }^{**}, \varvec{\varphi }^{**}}^{2, c_{\rho , O}, m} \left( \int _{1}^{\infty }\int _{0}^{\infty } \left[ \prod _{\rho =1}^{{\mathcal {U}}} \prod _{O=1}^{2} \Upsilon _{\varvec{\gamma }_{\rho , O},\varvec{\varphi }_{\rho , O}}(\varvec{ r}_{\rho , O})\,\, B_{\varvec{\gamma }_{\rho , O}}( {\textbf {y}}_{\rho , O})\,\, D_{\varvec{\varphi }_{\rho , O}}( {\textbf {y}}_{\rho , O}^{*})\,\,\right] \right. \right. \\&\quad \times \, \mu ^{-1} \eta ^{b_{1}-1} \,\exp \left[ - \sigma \mu - \left( \frac{\eta }{b_{2}}\right) ^{b_{1}} - \sum _{\rho =1}^{{\mathcal {U}}}\sum _{O=1}^{2} \frac{\mu ^{0-1}}{\eta } \Delta _{\varvec{\gamma }_{\rho , O},\varvec{\varphi }_{\rho , O}}( {\textbf {y}}_{\rho , O})\right] \Bigg )\,\text {d} \eta \,\,\text {d} \mu \Bigg ], \end{aligned} \end{aligned}$$
6. The general entropy LFU (GELFU) is defined as follows:
  $$\begin{aligned} {\mathcal {L}}({\hat{\Lambda }}_{G},\Lambda )\propto \left( \frac{{\hat{\Lambda }}_{G}}{\Lambda }\right) ^{\varrho }-\varrho \log \left[ \frac{{\hat{\Lambda }}_{G}}{\Lambda }\right] -1| \varrho \ne 0. \end{aligned}$$
  The BES of $\Lambda$, based on the GELFU, is given by
  51 $$\begin{aligned} \begin{aligned} {\hat{\Lambda }}_{G}=\left[ \text {E}(\Lambda ^{-\varrho })\right] ^{\frac{-1}{\varrho }}. \end{aligned}\end{aligned}$$
  From (35) and (51) the BESs of $\eta$ and $\mu$, based on the GELFU, are then given, respectively, by
  52 $$\begin{aligned}{} & {} \begin{aligned} {\hat{\eta }}_{G}&=\left\{ \Omega ^{-1} \sum _{{\varvec{P}}^{**}, \varvec{\gamma }^{**}, \varvec{\varphi }^{**}}^{2, c_{\rho , O}, m} \left( \int _{1}^{\infty }\int _{0}^{\infty } \left[ \prod _{\rho =1}^{{\mathcal {U}}} \prod _{O=1}^{2} \Upsilon _{\varvec{\gamma }_{\rho , O},\varvec{\varphi }_{\rho , O}}(\varvec{ r}_{\rho , O})\,\, B_{\varvec{\gamma }_{\rho , O}}( {\textbf {y}}_{\rho , O})\,\, D_{\varvec{\varphi }_{\rho , O}}( {\textbf {y}}_{\rho , O}^{*})\,\,\right] \right. \right. \\&\quad \times \mu ^{-1} \eta ^{b_{1}-\varrho -1} \,\exp \left[ - \left( \frac{\eta }{b_{2}}\right) ^{b_{1}} - \sum _{\rho =1}^{{\mathcal {U}}}\sum _{O=1}^{2} \frac{\mu ^{O-1}}{\eta } \Delta _{\varvec{\gamma }_{\rho , O},\varvec{\varphi }_{\rho , O}}( {\textbf {y}}_{\rho , O})\right] \Bigg )\,\text {d} \eta \,\,\text {d} \mu \Bigg \}^{\frac{-1}{\varrho }}, \end{aligned} \end{aligned}$$
  53 $$\begin{aligned}{} & {} \begin{aligned} {\hat{\mu }}_{G}&= \left\{ \Omega ^{-1} \sum _{{\varvec{P}}^{**}, \varvec{\gamma }^{**}, \varvec{\varphi }^{**}}^{2, c_{\rho , O}, m} \left( \int _{1}^{\infty }\int _{0}^{\infty } \left[ \prod _{\rho =1}^{{\mathcal {U}}} \prod _{O=1}^{2} \Upsilon _{\varvec{\gamma }_{\rho , O},\varvec{\varphi }_{\rho , O}}(\varvec{ r}_{\rho , O})\,\, B_{\varvec{\gamma }_{\rho , O}}( {\textbf {y}}_{\rho , O})\,\, D_{\varvec{\varphi }_{\rho , O}}( {\textbf {y}}_{\rho , O}^{*})\,\,\right] \right. \right. \\&\quad \times \, \left. \left. \mu ^{-1-\varrho } \eta ^{b_{1}-1} \,\exp \left[ - \left( \frac{\eta }{b_{2}}\right) ^{b_{1}} - \sum _{\rho =1}^{{\mathcal {U}}}\sum _{O=1}^{2} \frac{\mu ^{O-1}}{\eta } \Delta _{\varvec{\gamma }_{\rho , O},\varvec{\varphi }_{\rho , O}}( {\textbf {y}}_{\rho , O})\right] \right) \,\text {d} \eta \,\,\text {d} \mu \right\} ^{\frac{-1}{\varrho }}. \end{aligned} \end{aligned}$$

## Bayes estimation based on a SRSA under CSPALTE

Suppose that ${\textbf{x}}_{{SRSA}} = \{ \{ x_{{\rho ,1,1}} ,x_{{\rho ,1,2}} , \ldots ,x_{{\rho ,1,c_{{\rho ,1}} }} \} ,\{ x_{{\rho ,2,1}} ,x_{{\rho ,2,2}} , \ldots ,x_{{\rho ,2,c_{{\rho ,2}} }} \} \}$, $\rho =1, \dots , {\mathcal {U}}$, is a type-I hybrid censored ${\mathcal {U}}$-cycle SRSA under CSPALTE from a certain population with CDFU (5) and PDFU (6). Then, the LHF can be written as

54 $$\begin{aligned} \begin{aligned} {\mathfrak {L}}(\eta ,\mu ;\text {{x}})&\varpropto \prod _{\rho =1}^{{\mathcal {U}}} \left\{ \prod _{O=1}^{2} \left[ 1-H_{O}(x_{\rho ,O}^{*} )\right] ^{m-c_{\rho ,O}} \prod _{K=1}^{c_{\rho ,O}} h_{O}(x_{\rho ,O,k})\right\} \\&=\prod _{\rho =1}^{{\mathcal {U}}} \left\{ \prod _{O=1}^{2} \left( 1+ \exp \left[ {\frac{\mu ^{O-1} x_{\rho ,O}^{*}}{\eta }}\right] \right) ^{c_{\rho ,O}-m} \prod _{K=1}^{c_{\rho ,O}} \displaystyle \frac{ \mu ^{O-1} \exp \left[ {\frac{-\mu ^{O-1} x_{\rho ,O,k}}{\eta }}\right] }{\eta \left( 1+ \exp \left[ {\frac{-\mu ^{O-1} x_{\rho ,O,k}}{\eta }}\right] \right) ^{2}} \right\} , \end{aligned} \end{aligned}$$

where

55 $$\begin{aligned} x_{\rho ,O}^{*}=\left\{ \begin{array}{ll} x_{\rho ,O,s}, &{} \quad \text {if} \quad x_{\rho ,O, s} \le \tau _{O},\\ \tau _{O}, &{} \quad \text {if} \quad x_{\rho ,O, s} > \tau _{O}. \end{array}\right. \end{aligned}$$

From (34) to (54), the joint posterior density function of $\eta$ and $\mu$ can take the following form

56 $$\begin{aligned} \begin{aligned} \pi ^{**}(\eta ,\mu ;\text {{x}})&=\zeta ^{-1} \prod _{\rho =1}^{{\mathcal {U}}} \left\{ \prod _{O=1}^{2} \left( 1+ \exp \left[ {\frac{\mu ^{O-1} x_{\rho ,O}^{*}}{\eta }}\right] \right) ^{c_{\rho ,O}-m} \prod _{K=1}^{c_{\rho ,O}} \displaystyle \frac{ \mu ^{O-1} \exp \left[ {\frac{-\mu ^{O-1} x_{\rho ,O,k}}{\eta }}\right] }{\eta \left( 1+ \exp \left[ {\frac{-\mu ^{O-1} x_{\rho ,O,k}}{\eta }}\right] \right) ^{2}} \right\} \\&\quad \times \, \mu ^{-1} \eta ^{b_{1}-1} \, \exp \left[ {- \left( \frac{\eta }{b_{2}}\right) ^{b_{1}}}\right] ,\, \end{aligned} \end{aligned}$$

where

57 $$\begin{aligned} \begin{aligned} \zeta&=\int _{1}^{\infty }\int _{0}^{\infty } \prod _{\rho =1}^{{\mathcal {U}}} \left\{ \prod _{O=1}^{2} \left( 1+ \exp \left[ {\frac{\mu ^{O-1} x_{\rho ,O}^{*}}{\eta }}\right] \right) ^{c_{\rho ,O}-m} \prod _{K=1}^{c_{\rho ,O}} \displaystyle \frac{ \mu ^{O-1} \exp \left[ {\frac{-\mu ^{O-1} x_{\rho ,O,k}}{\eta }}\right] }{\eta \left( 1+ \exp \left[ {\frac{-\mu ^{O-1} x_{\rho ,O,k}}{\eta }}\right] \right) ^{2}} \right\} \\&\quad \times \, \mu ^{-1} \eta ^{b_{1}-1} \, \exp \left[ {- \left( \frac{\eta }{b_{2}}\right) ^{b_{1}}}\right] \,\text {d} \eta \,\,\text {d} \mu . \end{aligned} \end{aligned}$$

1. From (56) to (36), the BESs of $\eta$ and $\mu$, based on the SELFU, are given, respectively, by
  58 $$\begin{aligned}{} & {} \begin{aligned} {\hat{\eta }}_{S}&=\zeta ^{-1}\int _{1}^{\infty }\int _{0}^{\infty } \prod _{\rho =1}^{{\mathcal {U}}} \left\{ \prod _{O=1}^{2} \left( 1+ \exp \left[ {\frac{\mu ^{O-1} x_{\rho ,O}^{*}}{\eta }}\right] \right) ^{c_{\rho ,O}-m} \prod _{K=1}^{c_{\rho ,O}} \displaystyle \frac{ \mu ^{O-1} \exp \left[ {\frac{-\mu ^{O-1} x_{\rho ,O,k}}{\eta }}\right] }{\eta \left( 1+ \exp \left[ {\frac{-\mu ^{O-1} x_{\rho ,O,k}}{\eta }}\right] \right) ^{2}} \right\} \\&\quad \times \mu ^{-1} \eta ^{b_{1}} \, \exp \left[ {- \left( \frac{\eta }{b_{2}}\right) ^{b_{1}}}\right] \,\text {d} \eta \,\,\text {d} \mu , \end{aligned} \end{aligned}$$
  59 $$\begin{aligned}{} & {} \begin{aligned} {\hat{\mu }}_{S}&=\zeta ^{-1}\int _{1}^{\infty }\int _{0}^{\infty } \prod _{\rho =1}^{{\mathcal {U}}} \left\{ \prod _{O=1}^{2} \left( 1+ \exp \left[ {\frac{\mu ^{O-1} x_{\rho ,O}^{*}}{\eta }}\right] \right) ^{c_{\rho ,O}-m} \prod _{K=1}^{c_{\rho ,O}} \displaystyle \frac{ \mu ^{O-1} \exp \left[ {\frac{-\mu ^{O-1} x_{\rho ,O,k}}{\eta }}\right] }{\eta \left( 1+ \exp \left[ {\frac{-\mu ^{O-1} x_{\rho ,O,k}}{\eta }}\right] \right) ^{2}} \right\} \\&\quad \times \eta ^{b_{1}-1} \, \exp \left[ {- \left( \frac{\eta }{b_{2}}\right) ^{b_{1}}}\right] \,\text {d} \eta \,\,\text {d} \mu . \end{aligned} \end{aligned}$$
2. From (56) to (39), the BESs of $\eta$ and $\mu$, based on the WSLFU, are then given, respectively, by
  60 $$\begin{aligned}{} & {} \begin{aligned} {\hat{\eta }}_{WS}&=\left( \zeta ^{-1}\int _{1}^{\infty }\int _{0}^{\infty } \prod _{\rho =1}^{{\mathcal {U}}} \left\{ \prod _{O=1}^{2} \left( 1+ \exp \left[ {\frac{\mu ^{O-1} x_{\rho ,O}^{*}}{\eta }}\right] \right) ^{c_{\rho ,O}-m} \prod _{K=1}^{c_{\rho ,O}} \displaystyle \frac{ \mu ^{O-1} \exp \left[ {\frac{-\mu ^{O-1} x_{\rho ,O,k}}{\eta }}\right] }{\eta \left( 1+ \exp \left[ {\frac{-\mu ^{O-1} x_{\rho ,O,k}}{\eta }}\right] \right) ^{2}} \right\} \right. \\&\quad \times \left. \, \mu ^{-1} \eta ^{b_{1}-2} \, \exp \left[ {- \left( \frac{\eta }{b_{2}}\right) ^{b_{1}}}\right] \,\text {d} \eta \,\,\text {d} \mu \right) ^{-1}, \end{aligned} \end{aligned}$$
  61 $$\begin{aligned}{} & {} \begin{aligned} {\hat{\mu }}_{G}&=\left( \zeta ^{-1}\int _{1}^{\infty }\int _{0}^{\infty } \prod _{\rho =1}^{{\mathcal {U}}} \left\{ \prod _{O=1}^{2} \left( 1+ \exp \left[ {\frac{\mu ^{O-1} x_{\rho ,O}^{*}}{\eta }}\right] \right) ^{c_{\rho ,O}-m} \prod _{K=1}^{c_{\rho ,O}} \displaystyle \frac{ \mu ^{O-1} \exp \left[ {\frac{-\mu ^{O-1} x_{\rho ,O,k}}{\eta }}\right] }{\eta \left( 1+ \exp \left[ {\frac{-\mu ^{O-1} x_{\rho ,O,k}}{\eta }}\right] \right) ^{2}} \right\} \right. \\&\quad \times \mu ^{-2} \eta ^{b_{1}-1} \, \exp \left[ {- \left( \frac{\eta }{b_{2}}\right) ^{b_{1}}}\right] \,\text {d} \eta \,\,\text {d} \mu \Bigg )^{-1}. \end{aligned} \end{aligned}$$
3. From (56) to (42), the BESs of $\eta$ and $\mu$, based on the PRLFU, are then given, respectively, by
  62 $$\begin{aligned}{} & {} \begin{aligned} {\hat{\eta }}_{P}&=\left( \zeta ^{-1}\int _{1}^{\infty }\int _{0}^{\infty } \prod _{\rho =1}^{{\mathcal {U}}} \left\{ \prod _{O=1}^{2} \left( 1+ \exp \left[ {\frac{\mu ^{O-1} x_{\rho ,O}^{*}}{\eta }}\right] \right) ^{c_{\rho ,O}-m} \prod _{K=1}^{c_{\rho ,O}} \displaystyle \frac{ \mu ^{O-1} \exp \left[ {\frac{-\mu ^{O-1} x_{\rho ,O,k}}{\eta }}\right] }{\eta \left( 1+ \exp \left[ {\frac{-\mu ^{O-1} x_{\rho ,O,k}}{\eta }}\right] \right) ^{2}} \right\} \right. \\&\quad \times \, \left. \mu ^{-1} \eta ^{b_{1}+1} \, \exp \left[ {- \left( \frac{\eta }{b_{2}}\right) ^{b_{1}}}\right] \,\text {d} \eta \,\,\text {d} \mu \right) ^{\frac{1}{2}}, \end{aligned} \end{aligned}$$
  63 $$\begin{aligned}{} & {} \begin{aligned} {\hat{\mu }}_{P}&=\left( \zeta ^{-1}\int _{1}^{\infty }\int _{0}^{\infty } \prod _{\rho =1}^{{\mathcal {U}}} \left\{ \prod _{O=1}^{2} \left( 1+ \exp \left[ {\frac{\mu ^{O-1} x_{\rho ,O}^{*}}{\eta }}\right] \right) ^{c_{\rho ,O}-m} \prod _{K=1}^{c_{\rho ,O}} \displaystyle \frac{ \mu ^{O-1} \exp \left[ {\frac{-\mu ^{O-1} x_{\rho ,O,k}}{\eta }}\right] }{\eta \left( 1+ \exp \left[ {\frac{-\mu ^{O-1} x_{\rho ,O,k}}{\eta }}\right] \right) ^{2}} \right\} \right. \\&\quad \times \, \left. \mu \eta ^{b_{1}-1} \, \exp \left[ {- \left( \frac{\eta }{b_{2}}\right) ^{b_{1}}}\right] \,\text {d} \eta \,\,\text {d} \mu \right) ^{\frac{1}{2}}. \end{aligned} \end{aligned}$$
4. From (56) to (45), The BESs of $\eta$ and $\mu$, based on the LGLFU, are given, respectively, by
  64 $$\begin{aligned}{} & {} \begin{aligned} {\hat{\eta }}_{L}&=\exp \left[ \zeta ^{-1}\int _{1}^{\infty }\int _{0}^{\infty } \prod _{\rho =1}^{{\mathcal {U}}} \left\{ \prod _{O=1}^{2} \left( 1+ \exp \left[ {\frac{\mu ^{O-1} x_{\rho ,O}^{*}}{\eta }}\right] \right) ^{c_{\rho ,O}-m} \prod _{K=1}^{c_{\rho ,O}} \displaystyle \frac{ \mu ^{O-1} \exp \left[ {\frac{-\mu ^{O-1} x_{\rho ,O,k}}{\eta }}\right] }{\eta \left( 1+ \exp \left[ {\frac{-\mu ^{O-1} x_{\rho ,O,k}}{\eta }}\right] \right) ^{2}} \right\} \right. \\&\quad \times \, \left. \mu ^{-1} \log [\eta ] \eta ^{b_{1}-1} \, \exp \left[ {- \left( \frac{\eta }{b_{2}}\right) ^{b_{1}}}\right] \,\text {d} \eta \,\,\text {d} \mu \right] , \end{aligned} \end{aligned}$$
  65 $$\begin{aligned}{} & {} \begin{aligned} {\hat{\mu }}_{L}&=\exp \left[ \zeta ^{-1}\int _{1}^{\infty }\int _{0}^{\infty } \prod _{\rho =1}^{{\mathcal {U}}} \left\{ \prod _{O=1}^{2} \left( 1+ \exp \left[ {\frac{\mu ^{O-1} x_{\rho ,O}^{*}}{\eta }}\right] \right) ^{c_{\rho ,O}-m} \prod _{K=1}^{c_{\rho ,O}} \displaystyle \frac{ \mu ^{O-1} \exp \left[ {\frac{-\mu ^{O-1} x_{\rho ,O,k}}{\eta }}\right] }{\eta \left( 1+ \exp \left[ {\frac{-\mu ^{O-1} x_{\rho ,O,k}}{\eta }}\right] \right) ^{2}} \right\} \right. \\&\quad \times \, \left. \mu ^{-1} \log [\mu ] \eta ^{b_{1}-1} \, \exp \left[ {- \left( \frac{\eta }{b_{2}}\right) ^{b_{1}}}\right] \,\text {d} \eta \,\,\text {d} \mu \right] . \end{aligned} \end{aligned}$$
5. From (56) to (48), the BESs of $\eta$ and $\mu$, based on the LINEXLFU, are given, respectively, by
  66 $$\begin{aligned}{} & {} \begin{aligned} {\hat{\eta }}_{LI}&=\frac{-1}{\sigma }\log \left[ \zeta ^{-1}\int _{1}^{\infty }\int _{0}^{\infty } \prod _{\rho =1}^{{\mathcal {U}}} \left\{ \prod _{O=1}^{2} \left( 1+ \exp \left[ {\frac{\mu ^{O-1} x_{\rho ,O}^{*}}{\eta }}\right] \right) ^{c_{\rho ,O}-m} \prod _{K=1}^{c_{\rho ,O}} \displaystyle \frac{ \mu ^{O-1} \exp \left[ {\frac{-\mu ^{O-1} x_{\rho ,O,k}}{\eta }}\right] }{\eta \left( 1+ \exp \left[ {\frac{-\mu ^{O-1} x_{\rho ,O,k}}{\eta }}\right] \right) ^{2}} \right\} \right. \\&\quad \times \, \left. \mu ^{-1} \eta ^{b_{1}-1} \, \exp \left[ {-\sigma \eta - \left( \frac{\eta }{b_{2}}\right) ^{b_{1}}}\right] \,\text {d} \eta \,\,\text {d} \mu \right] . \end{aligned} \end{aligned}$$
  67 $$\begin{aligned}{} & {} \begin{aligned} {\hat{\mu }}_{LI}&=\frac{-1}{\sigma }\log \left[ \zeta ^{-1}\int _{1}^{\infty }\int _{0}^{\infty } \prod _{\rho =1}^{{\mathcal {U}}} \left\{ \prod _{O=1}^{2} \left( 1+ \exp \left[ {\frac{\mu ^{O-1} x_{\rho ,O}^{*}}{\eta }}\right] \right) ^{c_{\rho ,O}-m} \prod _{K=1}^{c_{\rho ,O}} \displaystyle \frac{ \mu ^{O-1} \exp \left[ {\frac{-\mu ^{O-1} x_{\rho ,O,k}}{\eta }}\right] }{\eta \left( 1+ \exp \left[ {\frac{-\mu ^{O-1} x_{\rho ,O,k}}{\eta }}\right] \right) ^{2}} \right\} \right. \\&\quad \times \, \left. \mu ^{-1} \eta ^{b_{1}-1} \, \exp \left[ {-\sigma \mu - \left( \frac{\eta }{b_{2}}\right) ^{b_{1}}}\right] \,\text {d} \eta \,\,\text {d} \mu \right] . \end{aligned} \end{aligned}$$
6. From (56) to (51), the BESs of $\eta$ and $\mu$, based on the GELFU, are then given, respectively, by
  68 $$\begin{aligned}{} & {} \begin{aligned} {\hat{\eta }}_{G}&=\left( \zeta ^{-1}\int _{1}^{\infty }\int _{0}^{\infty } \prod _{\rho =1}^{{\mathcal {U}}} \left\{ \prod _{O=1}^{2} \left( 1+ \exp \left[ {\frac{\mu ^{O-1} x_{\rho ,O}^{*}}{\eta }}\right] \right) ^{c_{\rho ,O}-m} \prod _{K=1}^{c_{\rho ,O}} \displaystyle \frac{ \mu ^{O-1} \exp \left[ {\frac{-\mu ^{O-1} x_{\rho ,O,k}}{\eta }}\right] }{\eta \left( 1+ \exp \left[ {\frac{-\mu ^{O-1} x_{\rho ,O,k}}{\eta }}\right] \right) ^{2}} \right\} \right. \\&\quad \times \, \left. \mu ^{-1} \eta ^{b_{1}-\varrho -1} \, \exp \left[ {- \left( \frac{\eta }{b_{2}}\right) ^{b_{1}}}\right] \,\text {d} \eta \,\,\text {d} \mu \right) ^{\frac{-1}{\varrho }}, \end{aligned} \end{aligned}$$
  69 $$\begin{aligned}{} & {} \begin{aligned} {\hat{\mu }}_{G}&=\left( \zeta ^{-1}\int _{1}^{\infty }\int _{0}^{\infty } \prod _{\rho =1}^{{\mathcal {U}}} \left\{ \prod _{O=1}^{2} \left( 1+ \exp \left[ {\frac{\mu ^{O-1} x_{\rho ,O}^{*}}{\eta }}\right] \right) ^{c_{\rho ,O}-m} \prod _{K=1}^{c_{\rho ,O}} \displaystyle \frac{ \mu ^{0-1} \exp \left[ {\frac{-\mu ^{O-1} x_{\rho ,O,k}}{\eta }}\right] }{\eta \left( 1+ \exp \left[ {\frac{-\mu ^{O-1} x_{\rho ,O,k}}{\eta }}\right] \right) ^{2}} \right\} \right. \\&\quad \times \, \left. \mu ^{-1-\varrho } \eta ^{b_{1}-1} \, \exp \left[ {- \left( \frac{\eta }{b_{2}}\right) ^{b_{1}}}\right] \,\text {d} \eta \,\,\text {d} \mu \right) ^{\frac{-1}{\varrho }}. \end{aligned} \end{aligned}$$

##  Real data set:Light-emitting diodes

Light-emitting diodes (LEDs) find widespread application in various semiconductor devices, particularly in television screens and color displays. These LEDs are composed of thinly layered semiconductor materials, featuring significant doping. The specific spectral wavelength emitted during forward-biasing of the LED is contingent upon the semiconductor material and the extent of doping. In this study, adopting the methodology delineated by Dey et al.^44^, we explore occurrences of failures both under normal operating conditions (sample size: 58) and accelerated stress conditions (sample size: 58) after 1000 hours, as summarized in Table 1.

**Figure 2:**
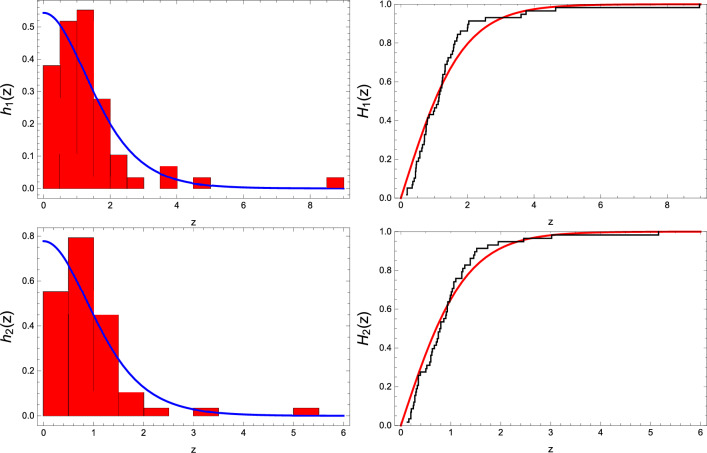
Comparative representation of histograms and empirical CDFUs against PDFUs and CDFUs of the half-logistic distribution, employing the provided data from Table 1 across two different stress levels.

Before delving further, we employ the statistic test of Kolmogorov-Smirnov (K-SM) along with the *p*-value for each stress level. The objective is to evaluate the appropriateness of fitting the data with the half-logistic distribution and its CDFU [see Eq. (5)]. The outcomes, presented in Table 2, affirm that CDFU (5) aptly captures the actual data at each stress level, given that all *p*-values surpass 0.05. This statistical fit is visually reinforced by plotting the empirical CDFU of the real data against the CDFU of the half-logistic distribution [see Eq. (5)], as depicted in Fig. 2.

We choose hyperparameter values of $b_{1}=1.15$, and $b_{2}=0.966$ to yield a population parameter value of ${\hat{\eta }}=0.9197$ using (32).

In the context of CSPALTE, we opt for five SRSAs, each comprising ten elements. These SRSAs are subsequently partitioned into two groups, each containing five elements, as delineated in the second and fourth columns of Table 3. Within each SRSA, the first and second sets of elements are selected from the data pertaining to standard and accelerated stresses, respectively. The RSSA technique is then applied to these SRSAs, resulting in the 1-cycle and 2-cycle RSSAs, which is presented in the third and fifth columns of Table 3. For both the 1-cycle and 2-cycle scenarios, we employ the type-I hybrid censoring procedure on the data originating from the SRSA (specifically, the first sample) and the associated RSSAs, all of which are detailed in Table 3.

Based on the SRSA and ORSSA data under CSPALTE, presented in Tables 4 and 5, BESs of $\eta$ and $\mu$ are computed using different LFUs, including SELFU, WSELFU, PRLFU, LGLFU, LINEXLFU, and GELFU, as summarized in Tables 6 and 7.

**Table 1:** LED failure data.

Standard stress	
0.18, 0.19, 0.19, 0.34, 0.36, 0.40, 0.44, 0.44, 0.45, 0.46, 0.47, 0.53, 0.57, 0.57, 0.63, 0.65, 0.70, 0.71, 0.71, 0.75, 0.76,	
0.76, 0.79, 0.80, 0.85, 0.98, 1.01, 1.07, 1.12, 1.14, 1.15, 1.17, 1.20, 1.23, 1.24, 1.25, 1.26, 1.32, 1.33, 1.33, 1.39, 1.42,	
1.50, 1.55, 1.58, 1.59, 1.62, 1.68, 1.70, 1.79, 2.00, 2.01, 2.04, 2.54, 3.61, 3.76, 4.65, 8.97	
Accelerated stress	
0.13, 0.16, 0.20, 0.20, 0.21, 0.25, 0.26, 0.28, 0.28, 0.30, 0.31, 0.33, 0.35, 0.35, 0.35, 0.39, 0.50, 0.52, 0.58, 0.60, 0.60,	
0.62, 0.63, 0.67, 0.71, 0.73, 0.75, 0.75, 0.78, 0.80, 0.80, 0.86, 0.90, 0.91, 0.93, 0.93, 0.94, 0.98, 0.99, 1.01, 1.03, 1.06,	
1.06, 1.10, 1.22, 1.22, 1.24, 1.28, 1.39, 1.39, 1.46, 1.48, 1.52, 1.74, 1.95, 2.46, 3.02, 5.16

**Table 2:** MLEs of the parameters $(\eta , \mu )$, K-SM statistic, and *p*-value.

Parameters	Stress	K-SM	*p-*value
${\hat{\eta }}=0.9197, {\hat{\mu }}= 1.4297$	Standard stress	0.1169	0.4059
	Accelerated stress	0.1025	0.5761

**Table 3:** The real SRSAs and RSSAs (1-cycle and 2-cycle).

	Standard stress						Accelerated stress					
$\rho$	SRSAs					RSSA	SRSAs					RSSA
1	**0.34**	0.44	0.76	1.17	3.76	**0.34**	**0.13**	0.62	0.78	1.22	5.16	**0.13**
	0.40	**0.63**	0.70	0.85	1.14	**0.63**	0.21	**0.73**	1.03	1.06	2.46	**0.73**
	0.19	1.23	**1.33**	1.33	1.62	**1.33**	0.20	0.20	**0.50**	0.60	0.80	**0.50**
	0.36	0.45	0.79	**1.20**	1.59	**1.20**	0.90	0.93	1.01	**1.46**	1.52	**1.46**
	0.53	1.25	1.26	1.39	**1.68**	**1.68**	0.31	0.35	0.71	0.94	**1.24**	**1.24**
2	**0.44**	0.47	0.75	0.76	1.79	**0.44**	**0.16**	0.33	0.75	0.98	0.99	**0.16**
	0.65	**0.71**	1.15	1.24	8.97	**0.71**	0.28	**0.52**	0.80	1.48	3.02	**0.52**
	0.57	1.50	**1.55**	1.70	2.04	**1.55**	0.35	0.63	**0.93**	1.06	1.74	**0.93**
	0.19	0.46	0.57	**0.80**	1.32	**0.80**	0.26	0.30	0.35	**0.58**	1.39	**0.58**
	0.18	0.71	0.98	2.01	**4.65**	**4.65**	0.25	0.39	0.86	1.22	**1.28**	**1.28**

Significant values are in bold.

**Table 4:** The real SRSAs (specifically, the first sample for each stress) under type-I hybrid censoring.

$\rho$	*m*	*s*	$(\tau _{1}, \tau _{2})$	Standard stress					Accelerated stress				
1	5	3	(1.5, 1.25)	0.34	0.44	0.76			0.13	0.62	0.78		
		4		0.34	0.44	0.76	1.17		0.13	0.62	0.78	1.22	
		5		0.34	0.44	0.76	1.17		0.13	0.62	0.78	1.22	
		5	$(\infty , \infty )$	0.34	0.44	0.76	1.17	3.76	0.13	0.62	0.78	1.22	5.16
2	5	3	(1.5, 1.25)	0.44	0.47	0.75			0.16	0.33	0.75		
		4		0.44	0.47	0.75	0.76		0.16	0.33	0.75	0.98	
		5		0.44	0.47	0.75	0.76		0.16	0.33	0.75	0.98	0.99
		5	$(\infty , \infty )$	0.44	0.47	0.75	0.76	1.79	0.16	0.33	0.75	0.98	0.99

**Table 5:** The real ORSSAs under type-I hybrid censoring.

$\rho$	*m*	*s*	$(\tau _{1}, \tau _{2})$	Standard stress					Accelerated stress				
1	5	3	(1.5, 1.25)	0.34	0.63	1.2			0.13	0.5	0.73		
		4		0.34	0.63	1.2	1.33		0.13	0.5	0.73	1.24	
		5		0.34	0.63	1.2	1.33		0.13	0.5	0.73	1.24	
		5	$(\infty , \infty )$	0.34	0.63	1.2	1.33	1.68	0.13	0.5	0.73	1.24	1.46
2	5	3	(1.5, 1.25)	0.44	0.71	0.8			0.16	0.52	0.58		
		4		0.44	0.71	0.8			0.16	0.52	0.58	0.93	
		5		0.44	0.71	0.8			0.16	0.52	0.58	0.93	
		5	$(\infty , \infty )$	0.44	0.71	0.8	1.55	4.65	0.16	0.52	0.58	0.93	1.28

**Table 6:** BESs of $\eta$ and $\mu$ based on 1-cycle SRSAs and ORSSAs.

				SRSA		ORSSA	
*m*	*s*	$(\tau _{1}, \tau _{2})$	LFU	${\hat{\eta }}$	${\hat{\mu }}$	${\hat{\eta }}$	${\hat{\mu }}$
5	3	(1.5, 1.25)	SE	1.00228	1.7881	1.15808	1.87269
			WSE	0.860143	1.56461	1.06948	1.66928
			PR	1.08895	1.9398	1.20965	1.99805
			LG	0.926202	1.66298	1.11151	1.76271
			LINEX	1.01154	1.8175	1.16428	1.8977
			$$\sigma =-0.1$$				
			LINEX	0.872416	1.47846	1.06249	1.5736
			$$\sigma =2.0$$				
			GE	0.936954	1.67998	1.11819	1.77815
			$$\varrho =-0.15$$				
			GE	0.753614	1.42995	0.997073	1.52727
			$$\varrho =3.0$$				
	4		SE	0.95718	1.66545	0.99752	1.57109
			WSE	0.84715	1.49041	0.94361	1.45972
			PR	1.02425	1.78724	1.02834	1.64158
			LG	0.89845	1.56734	0.96939	1.51083
			LINEX	0.96395	1.68729	1.00067	1.5827
			$$\sigma =-0.1$$				
			LINEX	0.85787	1.42732	0.94506	1.41754
			$$\sigma =2.0$$				
			GE	0.90676	1.58061	0.97345	1.51926
			$$\varrho =-0.15$$				
			GE	0.76269	1.38313	0.89797	1.37984
			$$\varrho =3.0$$				
	5		SE	1.00324	1.70583	1.01099	1.58509
			WSE	0.88903	1.51888	0.95673	1.47101
			PR	1.07224	1.83392	1.04197	1.65696
			LG	0.94243	1.60138	0.98269	1.52344
			LINEX	1.01053	1.72937	1.0142	1.59704
			$$\sigma =-0.1$$				
			LINEX	0.8965	1.44901	0.95758	1.42704
			$$\sigma =2.0$$				
			GE	0.95106	1.61557	0.98678	1.53207
			$$\varrho =-0.15$$				
			GE	0.80059	1.40345	0.91068	1.38888
			$$\varrho =3.0$$				
	5	$(\infty , \infty )$	SE	1.36848	1.45667	0.88076	1.50794
			WSE	1.24991	1.35462	0.84511	1.42166
			PR	1.43683	1.5286	0.90043	1.56047
			LG	1.30637	1.39973	0.86237	1.46186
			LINEX	1.37825	1.46777	0.88252	1.51616
			$$\sigma =-0.1$$				
			LINEX	1.22494	1.32341	0.84954	1.39001
			$$\sigma =2.0$$				
			GE	1.31531	1.40744	0.86505	1.46838
			$$\varrho =-0.15$$				
			GE	1.15147	1.28927	0.81358	1.35633
			$$\varrho =3.0$$

**Table 7:** BESs of $\eta$ and $\mu$ based on 2-cycle SRSAs and ORSSAs.

				SRSA		ORSSA	
*m*	*s*	$(\tau _{1}, \tau _{2})$	LFU	${\hat{\eta }}$	${\hat{\mu }}$	${\hat{\eta }}$	${\hat{\mu }}$
5	3	(1.5, 1.25)	SE	0.85864	1.57262	1.01046	1.71402
			WSE	0.78358	1.43778	0.96423	1.58884
			PR	0.90397	1.66561	1.03647	1.78779
			LG	0.81878	1.49762	0.98645	1.64774
			LINEX	0.8627	1.58821	1.01315	1.72721
			$$\sigma =-0.1$$				
			LINEX	0.79517	1.39106	0.96472	1.53024
			$$\sigma =2.0$$				
			GE	0.82444	1.50783	0.98993	1.65721
			$$\varrho =-0.15$$				
			GE	0.72439	1.35127	0.92436	1.49138
			$$\varrho =3.0$$				
	4		SE	0.7576	1.42795	0.99905	1.64598
			WSE	0.70864	1.3422	0.96361	1.55198
			PR	0.78647	1.48614	1.0185	1.69941
			LG	0.73182	1.38071	0.98079	1.59685
			LINEX	0.75985	1.43667	1.00102	1.65506
			$$\sigma =-0.1$$				
			LINEX	0.71995	1.31599	0.96416	1.50833
			$$\sigma =2.0$$				
			GE	0.73551	1.38718	0.98346	1.60395
			$$\varrho =-0.15$$				
			GE	0.66845	1.28428	0.93213	1.4744
			$$\varrho =3.0$$				
	5		SE	0.8136	1.58089	1.00967	1.62331
			WSE	0.75851	1.45729	0.9743	1.53277
			PR	0.84604	1.66203	1.02908	1.67493
			LG	0.78459	1.51322	0.99144	1.57595
			LINEX	0.81633	1.59444	1.01166	1.63196
			$$\sigma =-0.1$$				
			LINEX	0.76868	1.41179	0.9745	1.49188
			$$\sigma =2.0$$				
			GE	0.78874	1.52258	0.9941	1.58279
			$$\varrho =-0.15$$				
			GE	0.71348	1.37276	0.94286	1.45827
			$$\varrho =3.0$$				
	5	$(\infty , \infty )$	SE	0.96065	1.36994	0.95192	1.68596
			WSE	0.91011	1.30333	0.92716	1.60683
			PR	0.98927	1.41374	0.96499	1.72858
			LG	0.93437	1.3336	0.93932	1.64535
			LINEX	0.96347	1.37618	0.95318	1.69332
			$$\sigma =-0.1$$				
			LINEX	0.91311	1.28418	0.92862	1.56537
			$$\sigma =2.0$$				
			GE	0.93817	1.33864	0.94118	1.65131
			$$\varrho =-0.15$$				
			GE	0.8667	1.25644	0.90409	1.53636
			$$\varrho =3.0$$				

### Remark 1

In cases where the hyperparameters are not known, one approach is to employ the empirical Bayes method to estimate them based on historical samples^45^. Another option is to utilize the hierarchical Bayes method, where an appropriate prior for the hyperparameters is employed^46^. Also, the elicitation method can be considered an approach for determining the values of hyperparameters within a prior distribution for one or more parameters of the sampling distribution, see^47^ and the references therein.

## Simulation study

In this section, we embark on a comprehensive exploration within the CSPALTE framework. Our focus is on a rigorous comparative Monte Carlo simulation study that involves an in-depth examination of the ORSSA and SRSA techniques, specifically under the conditions of type-I hybrid censoring. Furthermore, we delve into the computation and comparison of BESs for both $\eta$ and $\mu$, employing both SLFU and ASLFU. The orchestrated Monte Carlo simulation procedure unfolds as follows:

1. Set values for $\eta , \mu , {\mathcal {U}}, m, s, \tau _{1}, \tau _{2}, \rho , \varrho ,$
2. Choose hyperparameter values $b_{1}$ and $b_{2}$ to derive the population parameter value $\eta$, using Eq. (32).
3. Consider two stress levels, standard and accelerated stresses. Generate a set of *m* SRSAs, each of size 2*m*. Split each SRSA into two equal-sized groups. Generate the first and second groups in each SRSA using CDFU (5).
4. Apply the recursive RSSA procedure, as described in section “RSSA under CSPALTE”, to the SRSAs obtained in Step 3 to obtain a 1-cycle RSSA.
5. Apply the technique of type-I hybrid censoring, as detailed in section “RSSA from CSPALTE under type-I hybrid censoring”, to the values of the SRSA (specifically, the first sample) and the RSSA obtained in Step 4.
6. To obtain ${\mathcal {U}}$-cycle type-I hybrid censored ORSSA, repeat Steps 3–5 a total of ${\mathcal {U}}$ times.
7. Calculate the BESs for the parameters $\eta$ and $\mu$ under different LFUs, including SELFU, WSELFU, PRLFU, LGLFU, LINEXLFU, and GELFU, based on the SRSAs and ORSSAs under type-I hybrid censoring.
8. Repeat the previously mentioned steps a total of ${\mathcal {D}}=1000$ times.
9. Using the relationships below, calculate the estimates mean, mean squared errors (MSERs), and relative absolute biases (RABIs) of $\hat{{\mathfrak {B}}}$ over ${\mathcal {D}}$ iterations.
  $$\begin{aligned} \begin{aligned} \overline{\widehat{{\mathfrak {B}}}}={\mathcal {D}}^{-1}{\sum _{O=1}^{{\mathcal {D}}}\hat{{\mathfrak {B}}}_{O}},\quad \text {MSER}(\hat{{\mathfrak {B}}})={\mathcal {D}}^{-1}{\sum _{O=1}^{{\mathcal {D}}}(\hat{{\mathfrak {B}}}_{O}-{\mathfrak {B}})^{2}},\quad \text {RABI}(\hat{{\mathfrak {B}}})={\mathcal {D}}^{-1}{\mathfrak {B}}^{-1}{\sum _{O=1}^{{\mathcal {D}}} |\hat{{\mathfrak {B}}}_{O}-{\mathfrak {B}}|}, \end{aligned} \end{aligned}$$
  where $\hat{{\mathfrak {B}}}$ represents an estimate of ${\mathfrak {B}}$.
10. As indicated in Step 9, calculate the estimated means of the parameters $\eta$ and $\mu$ along with associated RABIs and MSERs.
11. The following formulas should be used to calculate the mean of MSER (MMSER) as well as the mean of RABI (MRABI):
  $$\begin{aligned} \begin{aligned} \text {MMSER}=\frac{\text {MSER}({\hat{\eta }})+\text {MSER}({\hat{\mu }})}{2},\quad \quad \quad \quad \text {MRABI}=\frac{\text {RABI}({\hat{\eta }})+\text {RABI}({\hat{\mu }})}{2}. \end{aligned} \end{aligned}$$

Taking into account that the value of the population parameter is $\mu =1.5$ and the hyperparameter values are $b_1=1.9$ and $b_2=0.68$ to produce the population parameter value $\eta =0.6$. Tables 8, 9, and 10 give the computational results.

### Simulation results

The tabulated numerical outcomes in Tables 8, 9, and 10 affirm that the estimations derived from the ORSSA method exhibit greater efficiency compared to those obtained through the SRSA approach. This conclusion is substantiated by the following key points:

1. When the cycles number (${\mathcal {U}}$) monotonically increases, under fixed values of *m*, *s*, $\tau _{1}$, and $\tau _{2}$, we consistently observe that the MSERs, RABIs, MMSERs, and MRABIs of estimates obtained through the ORSSA procedure are consistently lower than those obtained through the SRSA procedure.
2. Keeping *m*, *s*, $\tau _{1}$, $\tau _{2}$, and ${\mathcal {U}}$ fixed, we find that the ORSSA procedure consistently outperforms the SRSA procedure, leading to lower MSERs, RABIs, MMSERs, and MRABIs of the estimates.
3. Notably, estimates obtained under LINEXLFU (at $\sigma =2.0$) and GELFU (at $\varrho =3.0$) tend to be superior to those under SELFU, WSELFU, PRLFU, and LGLFU. This superiority is evident in their lower MSERs, RABIs, MMSERs, and MRABIs.
4. When *s* is increased while maintaining *m*, $\tau _{1}$, $\tau _{2}$, and ${\mathcal {U}}$ fixed, we consistently observe a decrease in MSERs, RABIs, MMSERs, and MRABIs.
5. Similarly, when $(\tau _{1}, \tau _{2})$ is increased while maintaining *m*, *s*, and ${\mathcal {U}}$ fixed, we consistently observe a decrease in MSERs, RABIs, MMSERs, and MRABIs.

It’s important to note that the above trends hold true in most cases, with occasional exceptions likely resulting from data fluctuations.

## Conclusions, discussion, and some potential future points

In recent years, the ORSSA technique has garnered significant attention, primarily due to its efficiency in producing estimates compared to simple random sampling methods. This paper delves into the application of the ORSSA within the context of CSPALTEs when the lifetime of a unit exposed to use stress adheres to the half-logistic distribution. The study adopts type-I hybrid censoring and explores both SLFU and ASLFU to examine the Bayesian estimations of the parameters under consideration, comparing the performance of ORSSA and SRSA.

To substantiate the theoretical findings, actual datasets have been employed, offering practical insight into the presented results. Additionally, a comprehensive simulation study, complemented by numerical computations, has been executed to assess the efficacy of Bayesian estimations derived from ORSSA in contrast to SRSA. The numerical outcomes affirm the superiority of ordered ranked set sampling as a sampling technique, reinforcing its growing prominence in research and applications.

Additionally, the paper explores an innovative approach to Bayesian estimation in the context of CSPALTEs by combining ORSSA and type-I hybrid censoring. The study provides a valuable contribution to the field of reliability engineering and statistical methodology for ALTE, offering a robust framework for modeling and estimating the lifetime distribution of a product under different stress levels.

One of the significant highlights of this research is the integration of ORSSA and type-I hybrid censoring. The ORSSA is a non-traditional sampling technique that has gained attention due to its efficiency in reducing sampling costs while maintaining the accuracy of parameter estimates. By applying ordered ranked set sampling in combination with type-I hybrid censoring, the study harnesses the strengths of both methods, leading to improved estimation precision and cost-effectiveness. This integration is a novel approach and addresses the practical constraints often faced in ALTE scenarios. The use of Bayesian estimation techniques in this study is noteworthy. Bayesian methods offer a flexible and coherent framework for handling uncertainty and making inferences about the parameters of interest. The incorporation of prior information and the posterior distribution of parameters in the analysis allows for more robust and informative estimates. Bayesian estimation is especially advantageous when dealing with limited data, as is often the case in ALTE.

The proposed methodology has practical applications in various industries, including aerospace, automotive, electronics, and more, where reliability and product lifespan are critical factors. By improving the accuracy and efficiency of parameter estimation in CSALTEs experiments, this research provides a valuable tool for engineers and researchers seeking to optimize product design and assess the reliability of components and systems.

While this study successfully integrates ORSSA and type-I hybrid censoring for Bayesian estimation in CSPALTs, there are several avenues for future research. Further exploration of different censoring strategies, the impact of prior distributions on estimation accuracy, and the development of computational tools for practical implementation could enhance the applicability of the proposed methodology.

**Table 8:** BESs of $\eta$ and $\mu$ based on 1000 SRSAs and ORSSAs in a 1-cycle.

*m*	*s*	$(\tau _{1}, \tau _{2})$	LFU	SRSA				ORSSA			
				$\overline{{\hat{\eta }}}$	MSER(${\hat{\eta }}$)	RABI(${\hat{\eta }}$)	AMSER	$\overline{\check{\eta }}$	MSER($\check{\eta }$)	RABI($\check{\eta }$)	AMSER
				$\overline{{\hat{\mu }}}$	MSER(${\hat{\mu }}$)	RABI(${\hat{\mu }}$)	ARABI	$\overline{\check{\mu }}$	MSER($\check{\mu }$)	RABI($\check{\mu }$)	ARABI
5	3	(0.4, 0.3)	SE	0.74469	0.04333	0.29076	0.98683	0.74954	0.04392	0.28751	0.45334
				2.50156	1.93033	0.66846	0.47961	2.11289	0.86276	0.41995	0.35373
			WSE	0.65552	0.02412	0.21272	0.38049	0.67966	0.02542	0.21165	0.20384
				2.01396	0.73686	0.35796	0.28534	1.81391	0.38225	0.24857	0.23011
			PR	0.79058	0.05889	0.34515	1.45408	0.78691	0.0575	0.33927	0.65198
				2.80702	2.84928	0.87135	0.60825	2.30134	1.24646	0.53944	0.43936
			LG	0.69946	0.03179	0.24617	0.63173	0.71368	0.0333	0.24423	0.3071
				2.23526	1.23168	0.49447	0.37032	1.95034	0.58089	0.32289	0.28356
			LINEX	0.74825	0.04459	0.2953	1.15799	0.75248	0.04503	0.29167	0.51432
			$\sigma =-0.1$	2.59902	2.2714	0.73306	0.51418	2.16286	0.98361	0.4519	0.37178
			LINEX	0.68266	0.02544	0.22014	0.09689	0.69823	0.02735	0.22083	0.07607
			$\sigma =2.0$	1.71784	0.16835	0.17784	0.19899	1.6362	0.12479	0.15282	0.18682
			GE	0.70618	0.03327	0.25202	0.6781	0.71895	0.03472	0.25009	0.32605
			$\varrho =-0.15$	2.27242	1.32294	0.51826	0.38514	1.97303	0.61739	0.33596	0.29302
			GE	0.57391	0.01908	0.18697	0.12377	0.61791	0.01663	0.17363	0.0887
			$\varrho =3.0$	1.70305	0.22847	0.18966	0.18831	1.61082	0.16078	0.16217	0.1679
		(1.1, 0.9)	SE	0.72319	0.03629	0.25290	0.88847	0.68105	0.02205	0.19585	0.32505
				2.37639	1.74064	0.59316	0.42303	1.94739	0.62804	0.32719	0.26152
			WSE	0.65314	0.02438	0.20439	0.38096	0.63731	0.01630	0.16734	0.17358
				1.96942	0.73755	0.34368	0.27404	1.73933	0.33087	0.22498	0.19616
			PR	0.76072	0.04639	0.29411	1.25482	0.70527	0.02672	0.21766	0.43478
				2.62132	2.46325	0.75137	0.52274	2.07172	0.84284	0.39803	0.30785
			LG	0.68722	0.02907	0.22302	0.59825	0.65841	0.01864	0.17918	0.23918
				2.15753	1.16744	0.45601	0.33951	1.83665	0.45971	0.27018	0.22468
			LINEX	0.72597	0.0371	0.25607	1.02593	0.68275	0.02242	0.19755	0.35905
			$\sigma =-0.1$	2.45316	2.01477	0.6433	0.44969	1.97793	0.69568	0.34529	0.27142
			LINEX	0.67466	0.0245	0.20461	0.09955	0.65089	0.01652	0.16877	0.07162
			$\sigma =2.0$	1.70151	0.17460	0.18301	0.19381	1.60480	0.12673	0.1557	0.16224
			GE	0.6925	0.02998	0.22677	0.63702	0.66171	0.01908	0.18141	0.25068
			$\varrho =-0.15$	2.18849	1.24406	0.47500	0.35089	1.8524	0.48227	0.27792	0.22967
			GE	0.59144	0.02106	0.19569	0.13535	0.59936	0.01410	0.15593	0.09005
			$\varrho =3.0$	1.69021	0.24964	0.19632	0.19601	1.58244	0.16600	0.16662	0.16127
	4	(0.4, 0.3)	SE	0.75205	0.04442	0.29714	0.76908	0.75922	0.04629	0.30069	0.39038
				2.39546	1.49373	0.59803	0.44759	2.09432	0.73446	0.40626	0.35347
			WSE	0.66346	0.02368	0.21049	0.29438	0.68881	0.02631	0.2209	0.16515
				1.94332	0.56508	0.31106	0.26078	1.7982	0.30399	0.23547	0.22819
			PR	0.79759	0.06073	0.3552	1.16732	0.7968	0.06071	0.35252	0.57732
				2.68982	2.27391	0.79336	0.57428	2.2823	1.09394	0.52593	0.43923
			LG	0.70714	0.03211	0.24695	0.4846	0.72312	0.0349	0.25652	0.25645
				2.14542	0.93709	0.4357	0.34133	1.93287	0.47801	0.30871	0.28261
			LINEX	0.75563	0.04574	0.30202	0.90247	0.76222	0.04748	0.30493	0.44376
			$\sigma =-0.1$	2.48425	1.7592	0.65684	0.47943	2.14298	0.84003	0.43751	0.37122
			LINEX	0.68971	0.0256	0.22031	0.08201	0.70697	0.02856	0.23184	0.06611
			$\sigma =2.0$	1.68771	0.13843	0.15793	0.18912	1.63014	0.10366	0.14479	0.18831
			GE	0.71382	0.0337	0.25342	0.52072	0.72843	0.03642	0.26257	0.27356
			$\varrho =-0.15$	2.17988	1.00774	0.45764	0.35553	1.95536	0.5107	0.32188	0.29222
			GE	0.58212	0.01727	0.17832	0.10383	0.6264	0.01634	0.17573	0.06928
			$\varrho =3.0$	1.66498	0.19039	0.16433	0.17133	1.5997	0.12221	0.14979	0.16276
		(1.1, 0.9)	SE	0.72878	0.03931	0.26591	0.6766	0.68043	0.02229	0.19511	0.20666
				2.23372	1.31389	0.50289	0.3844	1.83955	0.39102	0.27405	0.23458
			WSE	0.66534	0.02594	0.21612	0.31286	0.64458	0.01613	0.16602	0.11927
				1.89876	0.59977	0.30767	0.26189	1.68116	0.22240	0.20125	0.18364
			PR	0.76287	0.04946	0.30274	0.94213	0.69999	0.02670	0.21510	0.26988
				2.43703	1.83480	0.63063	0.46668	1.93233	0.51307	0.32267	0.26889
			LG	0.69622	0.03155	0.23757	0.46874	0.66198	0.0188	0.17892	0.15718
				2.05368	0.90593	0.39544	0.31651	1.7559	0.29555	0.23358	0.20625
			LINEX	0.73135	0.04015	0.26890	0.77073	0.68183	0.02264	0.19667	0.22364
			$\sigma =-0.1$	2.29316	1.50131	0.54085	0.40488	1.86019	0.42463	0.28558	0.24113
			LINEX	0.68359	0.02667	0.21872	0.09703	0.65517	0.01671	0.16917	0.06258
			$\sigma =2.0$	1.67699	0.16739	0.17854	0.19863	1.58177	0.10844	0.15243	0.1608
			GE	0.70100	0.03257	0.24137	0.49645	0.66468	0.01927	0.18115	0.16381
			$\varrho =-0.15$	2.07911	0.96032	0.41033	0.32585	1.76787	0.30835	0.23916	0.21015
			GE	0.60902	0.02006	0.19107	0.12792	0.61274	0.01281	0.14895	0.07036
			$\varrho =3.0$	1.66292	0.23577	0.19163	0.19135	1.55680	0.12792	0.15913	0.15404
5	5	(0.4, 0.3)	SE	0.74835	0.04348	0.29033	0.73355	0.75001	0.04426	0.29015	0.3554
				2.38463	1.42363	0.59099	0.44066	2.07034	0.66654	0.39265	0.3414
			WSE	0.65975	0.02338	0.20904	0.26762	0.68032	0.02546	0.2142	0.14569
				1.93335	0.51186	0.30552	0.25728	1.77965	0.26593	0.22678	0.22049
			PR	0.79392	0.05945	0.34742	1.13136	0.78728	0.05799	0.34121	0.53362
				2.6783	2.20327	0.78582	0.56662	2.2558	1.00925	0.50999	0.4256
			LG	0.70342	0.03149	0.24266	0.4518	0.71426	0.03349	0.24728	0.22969
				2.1349	0.8721	0.42918	0.33592	1.91158	0.42589	0.29776	0.27252
			LINEX	0.7519	0.04476	0.2951	0.86527	0.75296	0.04539	0.29434	0.40466
			$\sigma =-0.1$	2.47335	1.68577	0.64977	0.47243	2.11775	0.76394	0.42284	0.35859
			LINEX	0.68627	0.02514	0.217	0.07771	0.69869	0.02746	0.22378	0.0601
			$\sigma =2.0$	1.68344	0.13027	0.15694	0.18697	1.61884	0.09275	0.14131	0.18255
			GE	0.71009	0.03303	0.24869	0.48728	0.71951	0.03492	0.25306	0.24561
			$\varrho =-0.15$	2.1693	0.94153	0.45102	0.34985	1.93365	0.4563	0.31053	0.2818
			GE	0.57852	0.01756	0.17912	0.09328	0.6187	0.0164	0.17322	0.06059
			$\varrho =3.0$	1.658	0.16901	0.16285	0.17098	1.58626	0.10478	0.14488	0.15905
		(1.1, 0.9)	SE	0.71912	0.03518	0.25307	0.48851	0.6874	0.02521	0.20917	0.20898
				2.14119	0.94184	0.44188	0.34747	1.85218	0.39274	0.28013	0.24465
			WSE	0.65747	0.02262	0.19979	0.21743	0.65186	0.01821	0.17704	0.1228
				1.83726	0.41223	0.26473	0.23226	1.69672	0.2274	0.20796	0.1925
			PR	0.75226	0.0448	0.29099	0.70039	0.70673	0.03008	0.22924	0.27089
				2.3299	1.35599	0.56085	0.42592	1.94282	0.5117	0.32892	0.27908
			LG	0.68747	0.02787	0.22246	0.33019	0.66913	0.02129	0.1916	0.1603
				1.97672	0.6325	0.34264	0.28255	1.7702	0.29932	0.23998	0.21579
			LINEX	0.72161	0.03596	0.25608	0.55629	0.68882	0.02561	0.21081	0.22535
			$\sigma =-0.1$	2.19281	1.07661	0.47491	0.3655	1.87231	0.42509	0.2916	0.2512
			LINEX	0.67539	0.02355	0.20448	0.07728	0.66198	0.0189	0.18095	0.06751
			$\sigma =2.0$	1.65117	0.13101	0.16086	0.18267	1.59769	0.11613	0.15824	0.16959
			GE	0.69212	0.02883	0.22658	0.35082	0.6718	0.02183	0.19408	0.16684
			$\varrho =-0.15$	1.99979	0.67281	0.35615	0.29137	1.78195	0.31186	0.24544	0.21976
			GE	0.60269	0.01731	0.17516	0.09231	0.62017	0.01413	0.15753	0.07383
			$\varrho =3.0$	1.62766	0.16731	0.16829	0.17172	1.57371	0.13353	0.16404	0.16078
		$(\infty , \infty )$	SE	0.69249	0.02684	0.21634	0.4609	0.65839	0.01669	0.16851	0.17125
				2.09837	0.89496	0.41543	0.31589	1.8028	0.3258	0.25294	0.21073
			WSE	0.6408	0.01952	0.18306	0.22016	0.6319	0.01344	0.15149	0.10967
				1.82377	0.42081	0.2607	0.22188	1.67332	0.2059	0.19649	0.17399
			PR	0.72058	0.03284	0.24312	0.64203	0.67252	0.01897	0.18003	0.21368
				2.26588	1.25123	0.51963	0.38137	1.87629	0.40839	0.28994	0.23498
			LG	0.66589	0.02248	0.19655	0.32223	0.64486	0.01486	0.15888	0.13695
				1.95087	0.62197	0.32921	0.26288	1.73515	0.25904	0.22184	0.19036
			LINEX	0.69449	0.02732	0.21842	0.51983	0.65936	0.01687	0.16942	0.18215
			$\sigma =-0.1$	2.1434	1.01234	0.44399	0.33121	1.8183	0.34743	0.26133	0.21538
			LINEX	0.657	0.01971	0.18387	0.07967	0.64047	0.01371	0.15285	0.06404
			$\sigma =2.0$	1.64829	0.13962	0.16277	0.17332	1.58977	0.11437	0.15591	0.15438
			GE	0.66978	0.02304	0.19912	0.34054	0.64685	0.01511	0.16021	0.14162
			$\varrho =-0.15$	1.97169	0.65805	0.34096	0.27004	1.74493	0.26814	0.22613	0.19317
			GE	0.59511	0.01702	0.17376	0.10029	0.60759	0.01165	0.14248	0.07208
			$\varrho =3.0$	1.62721	0.18356	0.17195	0.17285	1.56654	0.13252	0.16139	0.15194

**Table 9:** BESs of $\eta$ and $\mu$ based on 1000 SRSAs and ORSSAs in a 2-cycle.

*m*	*s*	$(\tau _{1}, \tau _{2})$	LFU	SRSA				ORSSA			
				$\overline{{\hat{\eta }}}$	MSER(${\hat{\eta }}$)	RABI(${\hat{\eta }}$)	AMSER	$\overline{\check{\eta }}$	MSER($\check{\eta }$)	RABI($\check{\eta }$)	AMSER
				$\overline{{\hat{\mu }}}$	MSER(${\hat{\mu }}$)	RABI(${\hat{\mu }}$)	ARABI	$\overline{\check{\mu }}$	MSER($\check{\mu }$)	RABI($\check{\mu }$)	ARABI
5	3	(0.4, 0.3)	SE	0.75691	0.04783	0.30158	0.5684	0.73078	0.03909	0.26872	0.23584
				2.19941	1.08898	0.47798	0.38978	1.90487	0.43258	0.30207	0.28539
			WSE	0.68639	0.02833	0.22714	0.25077	0.6804	0.02526	0.2126	0.12056
				1.86741	0.4732	0.28339	0.25526	1.70703	0.21587	0.20287	0.20773
			PR	0.7941	0.06176	0.34995	0.81645	0.75817	0.04864	0.30322	0.32515
				2.407	1.57114	0.60977	0.47986	2.02481	0.60166	0.37022	0.33672
			LG	0.72091	0.03674	0.26065	0.38292	0.70484	0.03136	0.23863	0.16901
				2.01918	0.72909	0.36893	0.31479	1.79901	0.30666	0.24629	0.24246
			LINEX	0.75986	0.049	0.30558	0.64907	0.73291	0.0399	0.27159	0.25872
			$\sigma =-0.1$	2.25772	1.24915	0.51547	0.41052	1.93232	0.47754	0.31825	0.29492
			LINEX	0.70512	0.03025	0.23647	0.08821	0.69311	0.0267	0.22057	0.06168
			$\sigma =2.0$	1.66328	0.14618	0.16827	0.20237	1.59049	0.09666	0.14467	0.18262
			GE	0.72622	0.03823	0.26633	0.40709	0.70864	0.03241	0.24282	0.17776
			$\varrho =-0.15$	2.0444	0.77595	0.38367	0.325	1.81399	0.32311	0.25387	0.24835
			GE	0.62265	0.01857	0.18339	0.10392	0.63604	0.01717	0.17363	0.06376
			$\varrho =3.0$	1.64228	0.18926	0.17765	0.18052	1.56111	0.11034	0.14948	0.16156
		(1.1, 0.9)	SE	0.69155	0.02718	0.21822	0.43612	0.65509	0.01469	0.15733	0.15237
				2.07046	0.84506	0.40071	0.30946	1.78413	0.29005	0.24491	0.20112
			WSE	0.64291	0.01964	0.1842	0.21724	0.62887	0.01172	0.14033	0.09672
				1.8181	0.41485	0.26279	0.22349	1.66059	0.18173	0.19233	0.16633
			PR	0.7181	0.0331	0.24335	0.5961	0.66936	0.01685	0.16932	0.19068
				2.22167	1.15911	0.49198	0.36767	1.85398	0.36452	0.27815	0.22374
			LG	0.66648	0.02276	0.19836	0.31131	0.64161	0.013	0.14778	0.12138
				1.93581	0.59986	0.3242	0.26128	1.7197	0.22977	0.21629	0.18204
			LINEX	0.69345	0.02766	0.22024	0.48799	0.65606	0.01486	0.15824	0.16186
			$\sigma =-0.1$	2.11056	0.94833	0.42564	0.32294	1.79865	0.30887	0.25251	0.20538
			LINEX	0.65775	0.01992	0.18564	0.08377	0.63729	0.01197	0.14205	0.05835
			$\sigma =2.0$	1.65111	0.14762	0.17056	0.1781	1.58166	0.10473	0.15428	0.14816
			GE	0.67014	0.02334	0.201	0.32796	0.64358	0.01322	0.14906	0.12561
			$\varrho =-0.15$	1.95493	0.63258	0.33472	0.26786	1.72902	0.23799	0.22032	0.18469
			GE	0.60013	0.01653	0.17044	0.10224	0.6054	0.01018	0.13255	0.06282
			$\varrho =3.0$	1.6316	0.18795	0.18053	0.17549	1.55778	0.11545	0.15913	0.14584
	4	(0.4, 0.3)	SE	0.74562	0.04325	0.28386	0.38967	0.72904	0.03855	0.26792	0.23067
				2.08942	0.7361	0.40358	0.34372	1.91481	0.42279	0.30683	0.28738
			WSE	0.67643	0.02541	0.21353	0.16136	0.67858	0.02482	0.21222	0.11665
				1.78846	0.29732	0.22926	0.22139	1.71627	0.20849	0.20463	0.20842
			PR	0.78225	0.05626	0.33198	0.582	0.75649	0.04805	0.302	0.31941
				2.28283	1.10775	0.52609	0.42903	2.03509	0.59077	0.37582	0.33891
			LG	0.71025	0.03303	0.24423	0.2533	0.70305	0.03086	0.23803	0.16447
				1.92471	0.47357	0.30422	0.27423	1.80861	0.29809	0.2498	0.24391
			LINEX	0.74849	0.04432	0.28771	0.44534	0.73117	0.03934	0.27077	0.25279
			$\sigma =-0.1$	2.1397	0.84636	0.43565	0.36168	1.94225	0.46623	0.32318	0.29698
			LINEX	0.69529	0.02722	0.22172	0.06253	0.69135	0.02627	0.22	0.06085
			$\sigma =2.0$	1.62086	0.09784	0.13939	0.18056	1.59926	0.09542	0.14567	0.18283
			GE	0.71547	0.03439	0.24968	0.27063	0.70685	0.03191	0.24223	0.17313
			$\varrho =-0.15$	1.94757	0.50687	0.31748	0.28358	1.82365	0.31436	0.25763	0.24993
			GE	0.6142	0.01695	0.17532	0.06679	0.63418	0.01686	0.17457	0.06085
			$\varrho =3.0$	1.58976	0.11662	0.14397	0.15964	1.56951	0.10485	0.1495	0.16204
		(1.1, 0.9)	SE	0.68549	0.02633	0.21007	0.31679	0.64655	0.01387	0.15469	0.09102
				1.95508	0.60725	0.33548	0.27278	1.68841	0.16816	0.19621	0.17545
			WSE	0.64307	0.01913	0.17958	0.17187	0.62588	0.01130	0.14064	0.06262
				1.75343	0.32462	0.23273	0.20615	1.59747	0.11393	0.16426	0.15245
			PR	0.70858	0.03165	0.23183	0.42171	0.65762	0.01561	0.16369	0.11046
				2.07491	0.81177	0.40427	0.31805	1.73877	0.20532	0.21729	0.19049
			LG	0.66366	0.02221	0.19269	0.23462	0.63598	0.01244	0.14698	0.07522
				1.84797	0.44704	0.27821	0.23545	1.64132	0.13800	0.17849	0.16273
			LINEX	0.68715	0.02677	0.21184	0.34844	0.6473	0.01401	0.15541	0.09522
			$\sigma =-0.1$	1.98436	0.67012	0.35305	0.28245	1.69797	0.17644	0.20065	0.17803
			LINEX	0.65574	0.01956	0.18139	0.07796	0.6325	0.01157	0.14222	0.04441
			$\sigma =2.0$	1.62127	0.13636	0.16321	0.1723	1.54309	0.07724	0.14133	0.14177
			GE	0.66686	0.02276	0.19502	0.24563	0.63753	0.01264	0.14805	0.07738
			$\varrho =-0.15$	1.86322	0.46850	0.28604	0.24053	1.64817	0.14212	0.18096	0.16450
			GE	0.60544	0.01557	0.16591	0.09192	0.60700	0.00975	0.13169	0.0453
			$\varrho =3.0$	1.59978	0.16826	0.17179	0.16885	1.51924	0.08085	0.14537	0.13853
5	5	(0.4, 0.3)	SE	0.74397	0.04461	0.2867	0.39106	0.72939	0.03797	0.26114	0.21212
				2.10062	0.73751	0.40916	0.34793	1.89593	0.38626	0.29432	0.27773
			WSE	0.67503	0.02676	0.21883	0.16068	0.67909	0.02438	0.20549	0.10523
				1.79584	0.29461	0.23066	0.22474	1.70098	0.18609	0.19509	0.20029
			PR	0.78047	0.05758	0.33398	0.58618	0.75676	0.04739	0.29623	0.29629
				2.2965	1.11478	0.53476	0.43437	2.01435	0.54519	0.36317	0.3297
			LG	0.70873	0.0344	0.24822	0.25318	0.70348	0.03036	0.23104	0.14981
				1.93379	0.47196	0.30706	0.27764	1.79157	0.26927	0.23845	0.23474
			LINEX	0.74682	0.04569	0.29051	0.44708	0.73152	0.03875	0.264	0.23258
			$\sigma =-0.1$	2.15161	0.84847	0.44202	0.36627	1.92261	0.4264	0.31028	0.28714
			LINEX	0.69379	0.02841	0.22579	0.0628	0.69186	0.02583	0.21335	0.05583
			$\sigma =2.0$	1.62647	0.09719	0.13934	0.18257	1.5884	0.08583	0.13978	0.17657
			GE	0.71393	0.03576	0.25348	0.27067	0.70727	0.03139	0.23524	0.15793
			$\varrho =-0.15$	1.95694	0.50558	0.32059	0.28703	1.80633	0.28447	0.24604	0.24064
			GE	0.61303	0.0182	0.1817	0.06626	0.63486	0.01651	0.16867	0.05425
			$\varrho =3.0$	1.59492	0.11432	0.1431	0.1624	1.55734	0.09199	0.14427	0.15647
		(1.1, 0.9)	SE	0.6913	0.02877	0.22032	0.24177	0.65114	0.01393	0.15029	0.0953
				1.91145	0.45476	0.30894	0.26463	1.69524	0.17667	0.19919	0.17474
			WSE	0.65003	0.02079	0.18793	0.12936	0.63099	0.01115	0.13547	0.06712
				1.72487	0.23794	0.21535	0.20164	1.60757	0.12309	0.16801	0.15174
			PR	0.71369	0.03445	0.24246	0.32559	0.66191	0.01577	0.15994	0.1143
				2.02227	0.61673	0.37192	0.30719	1.74356	0.21282	0.21987	0.1899
			LG	0.67009	0.02427	0.2024	0.17742	0.64084	0.0124	0.14215	0.07971
				1.81237	0.33058	0.25712	0.22976	1.64992	0.14703	0.18201	0.16208
			LINEX	0.69294	0.02925	0.22213	0.26416	0.65188	0.01407	0.15105	0.09946
			$\sigma =-0.1$	1.93705	0.49908	0.32413	0.27313	1.70444	0.18485	0.2035	0.17727
			LINEX	0.66194	0.02135	0.19059	0.06521	0.63732	0.01148	0.1374	0.04802
			$\sigma =2.0$	1.60878	0.10907	0.15559	0.17309	1.55412	0.08456	0.14477	0.14108
			GE	0.6732	0.02487	0.20485	0.18596	0.64236	0.01261	0.14326	0.08185
			$\varrho =-0.15$	1.82649	0.34705	0.2642	0.23453	1.65653	0.1511	0.18439	0.16382
			GE	0.61327	0.01643	0.1709	0.07024	0.61251	0.00937	0.12591	0.04936
			$\varrho =3.0$	1.58239	0.12406	0.16066	0.16578	1.53158	0.08935	0.14812	0.13701
		$(\infty , \infty )$	SE	0.66085	0.01695	0.16928	0.2036	0.62625	0.00837	0.11856	0.05873
				1.84791	0.39026	0.27451	0.2219	1.6181	0.1091	0.16094	0.13975
			WSE	0.6288	0.01333	0.1511	0.11886	0.61181	0.00743	0.11218	0.04484
				1.69156	0.22438	0.20075	0.17592	1.55028	0.08226	0.1431	0.12764
			PR	0.67819	0.01971	0.18379	0.26436	0.63379	0.00902	0.12318	0.06814
				1.93888	0.50901	0.32277	0.25328	1.65474	0.12726	0.17337	0.14827
			LG	0.6444	0.01485	0.15875	0.15576	0.61893	0.00784	0.11487	0.05103
				1.76551	0.29666	0.23392	0.19634	1.58328	0.09421	0.15107	0.13297
			LINEX	0.66204	0.01716	0.17038	0.2201	0.62673	0.00842	0.11892	0.06067
			$\sigma =-0.1$	1.86808	0.42303	0.28591	0.22815	1.62457	0.11291	0.1634	0.14116
			LINEX	0.63921	0.01355	0.15198	0.06246	0.61695	0.00751	0.11254	0.03526
			$\sigma =2.0$	1.59234	0.11137	0.15119	0.15159	1.51197	0.063	0.12926	0.1209
			GE	0.64681	0.01513	0.16008	0.16219	0.62001	0.00791	0.11536	0.05208
			$\varrho =-0.15$	1.77733	0.30925	0.23951	0.1998	1.58838	0.09625	0.15242	0.13389
			GE	0.59997	0.01174	0.14397	0.07076	0.59819	0.00693	0.10937	0.03639
			$\varrho =3.0$	1.56766	0.12977	0.15672	0.15035	1.48983	0.06584	0.13261	0.12099

**Table 10:** BESs of $\eta$ and $\mu$ based on 1000 SRSAs and ORSSAs in a 3-cycle.

*m*	*s*	$(\tau _{1}, \tau _{2})$	LFU	SRSA				ORSSA			
				$\overline{{\hat{\eta }}}$	MSER(${\hat{\eta }}$)	RABI(${\hat{\eta }}$)	AMSER	$\overline{\check{\eta }}$	MSER($\check{\eta }$)	RABI($\check{\eta }$)	AMSER
				$\overline{{\hat{\mu }}}$	MSER(${\hat{\mu }}$)	RABI(${\hat{\mu }}$)	ARABI	$\overline{\check{\mu }}$	MSER($\check{\mu }$)	RABI($\check{\mu }$)	ARABI
5	3	(0.4, 0.3)	SE	0.72946	0.03984	0.26981	0.3547	0.69984	0.02857	0.22194	0.15359
				2.03977	0.66956	0.37826	0.32403	1.79009	0.27861	0.23749	0.22971
			WSE	0.67155	0.02533	0.21241	0.16515	0.6612	0.0196	0.18282	0.0878
				1.78289	0.30498	0.23456	0.22349	1.64229	0.156	0.17437	0.1786
			PR	0.76049	0.05016	0.30636	0.50422	0.72095	0.03472	0.24614	0.20315
				2.19878	0.95828	0.47581	0.39108	1.87744	0.37158	0.28292	0.26453
			LG	0.69977	0.03162	0.23833	0.24398	0.67993	0.02357	0.2007	0.11583
				1.90115	0.45634	0.29767	0.268	1.71178	0.20809	0.20156	0.20113
			LINEX	0.73185	0.0407	0.27287	0.39777	0.70142	0.02908	0.22393	0.16528
			$\sigma =-0.1$	2.07989	0.75483	0.40343	0.33815	1.80835	0.30149	0.24745	0.23569
			LINEX	0.68718	0.02666	0.21903	0.07031	0.67153	0.02058	0.18818	0.05202
			$\sigma =2.0$	1.62909	0.11396	0.15138	0.18521	1.55658	0.08346	0.13654	0.16236
			GE	0.70413	0.03273	0.24274	0.25838	0.68284	0.02425	0.20369	0.12083
			$\varrho =-0.15$	1.92063	0.48404	0.30866	0.2757	1.72296	0.2174	0.20635	0.20502
			GE	0.61978	0.01769	0.17959	0.07621	0.62711	0.01422	0.15664	0.0535
			$\varrho =3.0$	1.60165	0.13473	0.1569	0.16824	1.52769	0.09278	0.14249	0.14957
		(1.1, 0.9)	SE	0.67464	0.0217	0.192	0.25311	0.63781	0.01085	0.13414	0.08993
				1.90322	0.48451	0.30814	0.25007	1.67911	0.16902	0.18999	0.16207
			WSE	0.63761	0.01635	0.16722	0.14133	0.61933	0.0092	0.12513	0.06449
				1.72494	0.2663	0.2193	0.19326	1.59378	0.11977	0.16057	0.14285
			PR	0.69482	0.02569	0.21043	0.33368	0.64771	0.012	0.14062	0.10699
				2.00782	0.64167	0.36551	0.28797	1.72596	0.20198	0.20901	0.17482
			LG	0.65558	0.01863	0.17778	0.18985	0.62836	0.00992	0.12903	0.07589
				1.80896	0.36107	0.25937	0.21857	1.63505	0.14185	0.17377	0.1514
			LINEX	0.67605	0.02202	0.19342	0.27593	0.63847	0.01094	0.13464	0.09376
			$\sigma =-0.1$	1.92757	0.52985	0.32227	0.25785	1.68791	0.17659	0.194	0.16432
			LINEX	0.64904	0.01669	0.16871	0.06921	0.62555	0.00935	0.1256	0.04598
			$\sigma =2.0$	1.60967	0.12173	0.15834	0.16353	1.54233	0.08262	0.13926	0.13243
			GE	0.65837	0.01904	0.17966	0.19834	0.62975	0.01005	0.12973	0.07782
			$\varrho =-0.15$	1.82244	0.37764	0.26616	0.22291	1.64148	0.14559	0.176	0.15287
			GE	0.60472	0.01376	0.15482	0.07886	0.60242	0.00829	0.12076	0.04834
			$\varrho =3.0$	1.58601	0.14397	0.16418	0.1595	1.51944	0.08838	0.14361	0.13219
	4	(0.4, 0.3)	SE	0.73609	0.04053	0.27292	0.29859	0.71087	0.03118	0.23063	0.17482
				1.98404	0.55666	0.34438	0.30865	1.8375	0.31846	0.26481	0.24772
			WSE	0.67865	0.02529	0.21184	0.13767	0.67128	0.02124	0.18706	0.09961
				1.74382	0.25006	0.21375	0.21279	1.68303	0.17798	0.19315	0.19011
			PR	0.76683	0.05121	0.31244	0.42795	0.73248	0.03791	0.25782	0.2304
				2.13351	0.80469	0.43476	0.3736	1.92812	0.42289	0.31329	0.28556
			LG	0.70666	0.03195	0.23963	0.20405	0.69048	0.02567	0.20696	0.13194
				1.85425	0.37616	0.27059	0.25511	1.75586	0.23821	0.2248	0.21588
			LINEX	0.73848	0.04142	0.27613	0.33391	0.71251	0.03174	0.23283	0.18787
			$\sigma =-0.1$	2.02049	0.62639	0.36698	0.32155	1.85685	0.34399	0.2757	0.25426
			LINEX	0.69378	0.02687	0.21988	0.06264	0.68154	0.02234	0.19345	0.05872
			$\sigma =2.0$	1.60474	0.09841	0.14297	0.18143	1.5897	0.09509	0.14789	0.17067
			GE	0.71098	0.03311	0.24436	0.21628	0.69346	0.02643	0.21027	0.13765
			$\varrho =-0.15$	1.87247	0.39945	0.28058	0.26247	1.76753	0.24887	0.23023	0.22025
			GE	0.62719	0.01697	0.17521	0.06438	0.63631	0.0151	0.15841	0.05893
			$\varrho =3.0$	1.57478	0.1118	0.14909	0.16215	1.56205	0.10277	0.15174	0.15508
		(1.1, 0.9)	SE	0.67373	0.02085	0.18972	0.16787	0.63684	0.00969	0.12627	0.06412
				1.81289	0.31489	0.25767	0.2237	1.62603	0.11855	0.16526	0.14576
			WSE	0.64163	0.01566	0.16562	0.09871	0.62233	0.00821	0.11701	0.04967
				1.6694	0.18176	0.19336	0.17949	1.56166	0.09114	0.14839	0.1327
			PR	0.69111	0.02449	0.20618	0.21768	0.6445	0.01065	0.13225	0.07364
				1.89586	0.41086	0.30042	0.2533	1.66066	0.13663	0.17659	0.15442
			LG	0.65725	0.01793	0.17622	0.12876	0.62946	0.00888	0.12119	0.05619
				1.73745	0.23958	0.22194	0.19908	1.59303	0.10349	0.15593	0.13856
			LINEX	0.67496	0.02114	0.19103	0.18019	0.63735	0.00977	0.12672	0.06611
			$\sigma =-0.1$	1.8306	0.33924	0.26745	0.22924	1.63219	0.12246	0.16761	0.14717
			LINEX	0.65131	0.01616	0.16782	0.05669	0.62718	0.00841	0.11828	0.03922
			$\sigma =2.0$	1.58061	0.09723	0.15019	0.159	1.52422	0.07003	0.13427	0.12627
			GE	0.65966	0.01833	0.17806	0.13401	0.63055	0.00899	0.12189	0.05728
			$\varrho =-0.15$	1.74829	0.24969	0.22683	0.20245	1.59788	0.10557	0.15722	0.13955
			GE	0.61282	0.01273	0.15121	0.05988	0.6088	0.00723	0.11108	0.0403
			$\varrho =3.0$	1.55443	0.10703	0.15458	0.15289	1.50377	0.07337	0.13796	0.12452
5	5	(0.4, 0.3)	SE	0.7321	0.04139	0.27423	0.33221	0.70779	0.03051	0.22841	0.1588
				2.02323	0.62303	0.37042	0.32233	1.80185	0.2871	0.24545	0.23693
			WSE	0.67476	0.02643	0.21422	0.15677	0.66867	0.02078	0.1861	0.0906
				1.77469	0.28711	0.23415	0.22418	1.65265	0.16042	0.17953	0.18282
			PR	0.7628	0.05191	0.31199	0.47151	0.72913	0.0371	0.25475	0.20986
				2.17695	0.89111	0.46333	0.38766	1.8898	0.38262	0.29143	0.27309
			LG	0.70272	0.03296	0.24223	0.22957	0.68764	0.02511	0.2055	0.11975
				1.88918	0.42618	0.29376	0.26799	1.72287	0.21438	0.20843	0.20696
			LINEX	0.73448	0.04228	0.27732	0.37086	0.70941	0.03105	0.23056	0.17072
			$\sigma =-0.1$	2.0614	0.69944	0.39418	0.33575	1.82033	0.31038	0.25554	0.24305
			LINEX	0.68998	0.02782	0.22255	0.07022	0.67885	0.02186	0.19227	0.05418
			$\sigma =2.0$	1.62659	0.11262	0.15483	0.18869	1.56513	0.0865	0.13939	0.16583
			GE	0.70703	0.03409	0.24681	0.24289	0.69059	0.02585	0.2087	0.12492
			$\varrho =-0.15$	1.90803	0.45168	0.30415	0.27548	1.73414	0.22399	0.2135	0.2111
			GE	0.62343	0.01832	0.17805	0.07459	0.63411	0.01478	0.15846	0.05463
			$\varrho =3.0$	1.59863	0.13086	0.15917	0.16861	1.53657	0.09449	0.14403	0.15125
		(1.1, 0.9)	SE	0.67382	0.02053	0.18424	0.15254	0.63514	0.00915	0.12008	0.05763
				1.79266	0.28455	0.24298	0.21361	1.61539	0.1061	0.15764	0.13886
			WSE	0.64284	0.01531	0.1585	0.09193	0.62129	0.00775	0.11101	0.04532
				1.65798	0.16854	0.18515	0.17182	1.55511	0.08288	0.14268	0.12684
			PR	0.69057	0.02414	0.20111	0.19603	0.64243	0.01005	0.12566	0.06577
				1.87022	0.36792	0.28246	0.24179	1.64771	0.12149	0.16783	0.14675
			LG	0.65792	0.01762	0.17002	0.11831	0.6281	0.00839	0.11514	0.05086
				1.72195	0.21901	0.21088	0.19045	1.58452	0.09333	0.14929	0.13221
			LINEX	0.67501	0.02082	0.18558	0.16304	0.63562	0.00922	0.12051	0.05929
			$\sigma =-0.1$	1.80887	0.30525	0.25173	0.21865	1.62106	0.10936	0.15974	0.14012
			LINEX	0.6521	0.01584	0.16174	0.05485	0.62594	0.00794	0.1124	0.03658
			$\sigma =2.0$	1.57538	0.09387	0.1462	0.15397	1.52059	0.06521	0.13053	0.12146
			GE	0.66026	0.01801	0.17197	0.12292	0.62914	0.00849	0.11583	0.05179
			$\varrho =-0.15$	1.73213	0.22782	0.21523	0.1936	1.58905	0.09509	0.15041	0.13312
			GE	0.61494	0.01227	0.14324	0.05757	0.60833	0.00682	0.1052	0.0374
			$\varrho =3.0$	1.54921	0.10287	0.15086	0.14705	1.50064	0.06798	0.1346	0.1199
		$(\infty , \infty )$	SE	0.64058	0.01239	0.14563	0.1132	0.61733	0.00566	0.09852	0.03985
				1.72158	0.21401	0.2154	0.18052	1.57893	0.07405	0.14034	0.11943
			WSE	0.61765	0.01042	0.13457	0.07415	0.60729	0.0052	0.09536	0.03317
				1.61395	0.13788	0.17503	0.1548	1.53062	0.06113	0.12951	0.11243
			PR	0.65284	0.01385	0.15401	0.1404	0.62251	0.00598	0.10086	0.04429
				1.78214	0.26694	0.242	0.198	1.60434	0.0826	0.14741	0.12414
			LG	0.62886	0.01125	0.13912	0.09136	0.61226	0.00541	0.09667	0.03617
				1.66554	0.17146	0.19306	0.16609	1.55435	0.06693	0.13433	0.1155
			LINEX	0.64139	0.0125	0.14629	0.11948	0.61766	0.00569	0.0987	0.04072
			$\sigma =-0.1$	1.73354	0.22645	0.22125	0.18377	1.5832	0.07575	0.14175	0.12023
			LINEX	0.62543	0.01053	0.13483	0.04851	0.61098	0.00525	0.09537	0.02838
			$\sigma =2.0$	1.54867	0.0865	0.14629	0.14056	1.50416	0.05151	0.12079	0.10808
			GE	0.63058	0.0114	0.13996	0.09432	0.61301	0.00544	0.0969	0.03667
			$\varrho =-0.15$	1.67366	0.17724	0.19614	0.16805	1.55798	0.06791	0.13517	0.11603
			GE	0.59667	0.00951	0.13043	0.051	0.59766	0.00495	0.0941	0.02901
			$\varrho =3.0$	1.52374	0.09249	0.15093	0.14068	1.48576	0.05306	0.12342	0.10876

Below, we provide some relevant potential future point:

1. Investigate the applicability of Bayesian estimation in the context of other hybrid censoring schemes beyond type-I. Explore how the proposed methodology can be adapted or extended to accommodate different censoring structures, such as type-II or generalized hybrid censoring, to enhance its versatility and generalizability.
2. Explore the incorporation of dynamic stress levels in CSPALTEs. Investigate Bayesian estimation techniques that can effectively handle scenarios where stress levels change over time, allowing for a more realistic representation of the dynamic nature of stress conditions in practical applications.
3. Assess the robustness of the Bayesian estimation approach to various model assumptions. Investigate the impact of deviations from assumed distributional forms for the lifetime data and explore methods to enhance the methodology’s resilience to model misspecifications.
4. Further delve into the incorporation of informative prior distributions. Explore how prior knowledge about the system or product under study can be effectively integrated into the Bayesian framework, providing more informed and accurate estimates of the parameters of interest.
5. Conduct comparative studies with alternative estimation approaches in the context of CSPALTs. Compare the performance of the Bayesian methodology with frequentist counterparts or other Bayesian methods to identify strengths, limitations, and areas for improvement.
6. Extend the simulation studies to investigate the performance of the proposed Bayesian estimation method under conditions of small sample sizes. Assess the accuracy and reliability of parameter estimates when data availability is limited, which is often the case in real-world applications.
7. Explore the practical implementation and efficacy of the proposed Bayesian estimation approach in various industries. Consider applications in fields such as automotive, aerospace, electronics, and healthcare to validate the method’s utility and performance across diverse domains.
8. Consider the development of user-friendly software tools that implement the Bayesian estimation methodology for CSPALTEs. Facilitate broader adoption and application of the proposed approach by providing accessible computational tools for practitioners and researchers.
9. Investigate Bayesian estimation techniques that can accommodate scenarios involving multiple failure modes. Extend the methodology to handle complex systems where different failure mechanisms contribute to the overall reliability, providing a more comprehensive analysis.
10. Explore the integration of Bayesian estimation with reliability growth models. Investigate how the proposed methodology can be synergistically employed with models that capture the improvement in reliability over time, offering insights into the system’s evolving performance.

## Data Availability

All data generated or analysed during this study are included in this article.
